# *Orientia tsutsugamushi* Nucleomodulin Ank13 Exploits the RaDAR Nuclear Import Pathway To Modulate Host Cell Transcription

**DOI:** 10.1128/mBio.01816-21

**Published:** 2021-08-03

**Authors:** Haley E. Adcox, Amanda L. Hatke, Shelby E. Andersen, Sarika Gupta, Nathan B. Otto, Mary M. Weber, Richard T. Marconi, Jason A. Carlyon

**Affiliations:** a Department of Microbiology and Immunology, Virginia Commonwealth University Medical Center, School of Medicine, Richmond, Virginia, USA; b Department of Microbiology and Immunology, University of Iowa Health Care, Carver College of Medicine, Iowa City, Iowa, USA; Yale University School of Medicine

**Keywords:** *Orientia tsutsugamushi*, *Rickettsia*, ankyrin repeat, bacterial effector, intracellular bacterium, nucleomodulin

## Abstract

Orientia tsutsugamushi is the etiologic agent of scrub typhus, the deadliest of all diseases caused by obligate intracellular bacteria. Nucleomodulins, bacterial effectors that dysregulate eukaryotic transcription, are being increasingly recognized as key virulence factors. How they translocate into the nucleus and their functionally essential domains are poorly defined. We demonstrate that Ank13, an O. tsutsugamushi effector conserved among clinical isolates and expressed during infection, localizes to the nucleus in an importin β1-independent manner. Rather, Ank13 nucleotropism requires an isoleucine at the thirteenth position of its fourth ankyrin repeat, consistent with utilization of eukaryotic RaDAR (RanGDP-ankyrin repeats) nuclear import. RNA-seq analyses of cells expressing green fluorescent protein (GFP)-tagged Ank13, nucleotropism-deficient Ank13_I127R_, or Ank13ΔF-box, which lacks the F-box domain essential for interacting with SCF ubiquitin ligase, revealed Ank13 to be a nucleomodulin that predominantly downregulates transcription of more than 2,000 genes. Its ability to do so involves its nucleotropism and F-box in synergistic and mutually exclusive manners. Ank13 also acts in the cytoplasm to dysregulate smaller cohorts of genes. The effector’s toxicity in yeast heavily depends on its F-box and less so on its nucleotropism. Genes negatively regulated by Ank13 include those involved in the inflammatory response, transcriptional control, and epigenetics. Importantly, the majority of genes that GFP-Ank13 most strongly downregulates are quiescent or repressed in O. tsutsugamushi-infected cells when Ank13 expression is strongest. Ank13 is the first nucleomodulin identified to coopt RaDAR and a multifaceted effector that functions in the nucleus and cytoplasm via F-box-dependent and -independent mechanisms to globally reprogram host cell transcription.

## INTRODUCTION

Nucleomodulins are an emerging family of bacterial effectors that traffic into the nucleus to selectively control gene expression and thereby regulate eukaryotic cellular processes. Nearly all known nucleomodulins are deployed by intracellular bacteria (1, 2). Despite their importance as intracellular microbial virulence factors, how they traffic into the nucleus, their functionally essential domains and residues, and the genes that they dysregulate are poorly defined. Hence, characterizing nucleomodulins stands to reveal novel mechanisms by which intracellular bacteria remodel their host cells into permissive niches.

Orientia tsutsugamushi is a mite-transmitted obligate intracellular bacterium and the leading cause of scrub typhus, a severe infection prevalent in the Asia-Pacific region, where approximately one million new cases occur annually (3, 4). Locally acquired cases in the Middle East and South America, along with numerous travel-acquired cases, signify the disease as a global health threat (4–8). Acute symptoms include fever, headache, rash, and lymphadenopathy. If antibiotic therapy is delayed, scrub typhus can progress to pneumonitis, respiratory distress, meningitis, systemic vascular collapse, shock, organ failure, and death (3, 4). O. tsutsugamushi primarily invades monocytes, macrophages, and dendritic cells at the mite feeding site and disseminates via the lymphatics to invade endothelial cells of multiple organs (9). Within host cells, the bacterium replicates in the cytosol. While a type 1 inflammatory response is critical for both clearing and mediating immunopathological damage associated with scrub typhus (10–15), O. tsutsugamushi counteracts it and other host defense pathways (16–23), which likely contributes to its ability to successfully colonize host cells. The responsible bacterial factors and their mechanisms of action are largely unknown.

The 30- to 33-residue ankyrin repeat (AR) is one of the most common protein-protein interaction motifs in nature and a ubiquitous structural motif across the tree of life (24, 25). Analysis of more than 1,900 bacterial species revealed that obligate intracellular bacteria have the highest percentage of AR-containing proteins (Anks) within their proteomes, reflective of their prominence as interfaces with eukaryotic processes (24). Approximately 2.4% of the O. tsutsugamushi Ikeda strain genome encodes Anks, a percentage rivaling that of eukaryotes (24, 26). Specific O. tsutsugamushi Anks have been linked to the pathogen’s abilities to impair nuclear factor kappa-light-chain-enhancer of activated B cells (NF-κB) nuclear import, inhibit the secretory pathway, and degrade elongation factor 1α (16, 27, 28). O. tsutsugamushi Anks are type 1 secretion system effectors and are transcriptionally expressed during infection of mammalian cells (29). They share a two-dimensional architecture, having an N-terminal domain comprised of various numbers of tandemly arranged ARs (26, 29). Most also carry a C-terminal domain that mimics the eukaryotic F-box, which interacts with Skp1 (S-phase kinase-associated protein 1) of SCF E3 ubiquitin ligase complexes (26, 28, 30, 31). The SCF complex consists of Skp1, cullin-1 (Cul1), ring box (Rbx1), and an additional adaptor protein that carries an F-box (31). For O. tsutsugamushi and other microbial F-box-containing Anks, the prevailing model is that the AR domain binds a host cell target protein while the F-box binds Skp1 to nucleate the SCF complex, thereby orchestrating polyubiquitination of the AR-bound target and its degradation in the 26S proteasome (28, 30, 32–38).

Functions of most O. tsutsugamushi Anks remain to be determined. Our previous qualitative screen of the subcellular locations of the distinguishable 19 O. tsutsugamushi strain Ikeda Anks when ectopically expressed revealed that GFP- or Flag-tagged Ank13 (OTT_RS04140) was the only one to robustly accumulate in the nucleus whether the fusion tag was N or C terminal (29). Since this result is consistent with those observed for other pathogens’ nucleomodulins (39, 40), we examined here whether Ank13 is a nucleomodulin. Our findings establish Ank13 as a *bona fide* nucleomodulin and the first example of an effector that exploits the eukaryotic RaDAR (RanGDP-ankyrin repeats) nuclear import pathway. It downregulates cohorts of host genes, including those involved in transcriptional control, mRNA stability, epigenetic regulation, cell cycle control, and the inflammatory response. Its nucleomodulin ability and toxicity in yeast are dependent on both its nucleotropism and F-box. Many genes that ectopically expressed GFP-Ank13 downregulates are also repressed in O. tsutsugamushi-infected cells at a time point coincident with bacterial Ank13 expression. This study advances understanding of how O. tsutsugamushi transcriptionally regulates diverse host cellular processes, identifies a novel mechanism of nucleomodulin nuclear translocation, and underscores the contribution of the bacterial F-box as a eukaryotic transcriptional modulator.

## RESULTS

### Ank13 is conserved among *O. tsutsugamushi* isolates from scrub typhus patients.

**FIG 1:**
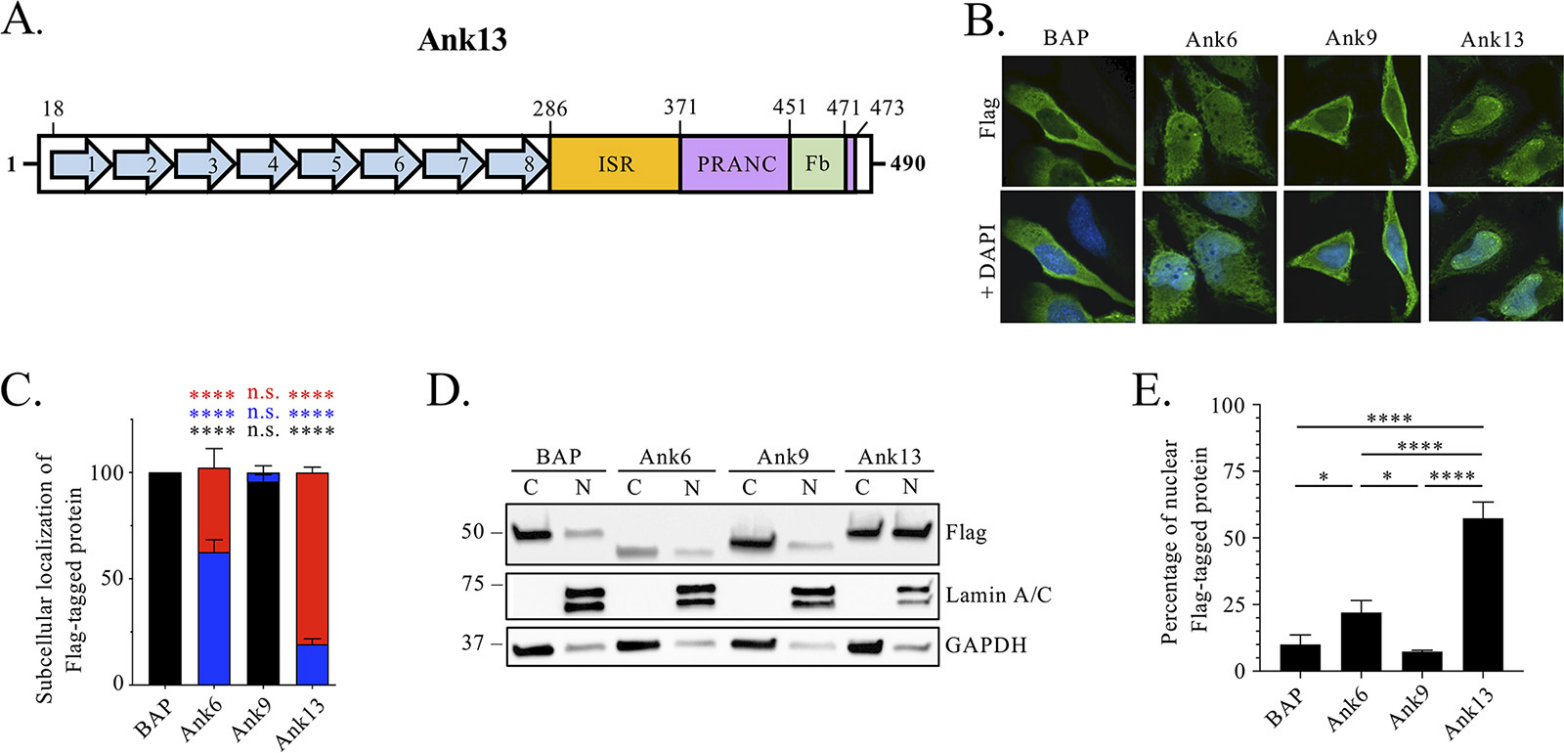
Ank13 is nucleotropic. (A) Schematic of Ank13 depicting its eight tandemly arranged ankyrin repeats (blue arrows), ISR (orange), and F-box (Fb; green). The Fb occurs as part of a larger encompassing PRANC domain (purple). Amino acids that constitute each domain are indicated. (B to E) Flag-Ank13 predominantly localizes to the nucleus. Transfected HeLa cells expressing Flag-tagged BAP, Ank6, Ank9, or Ank13 were examined by immunofluorescence microscopy (B and C) and Western blot analysis of nuclear [N] and cytoplasmic [C] fractions (D and E). (B) Representative images of fixed cells immunolabeled with Flag antibody and stained with DAPI. (C) One hundred cells were examined per condition in triplicate to quantify the means ± the standard deviations (SD) for the percentages of cells exhibiting Flag immunosignal localization, which was scored as being exclusively cytoplasmic (black), throughout the cell (blue), or exclusively in the nucleus (red). Two-way ANOVA with Dunnett’s correction determined significance between subcellular locations of Flag-tagged proteins compared to Flag-BAP. The data are representative of three experiments with similar results. (D) Fractions were probed with lamin A/C and GAPDH antibodies to verify fraction purity and Flag antibody to determine localization of Flag-tagged protein. (E) The nuclear densitometric value was divided by the sum of nuclear and cytoplasmic densitometric values for Flag-tagged proteins in panel D. The quotient was multiplied by 100 to yield the percentage of Flag-tagged protein in the nucleus. Data presented are percentages (means ± the SD) of Flag-tagged proteins exhibiting nuclear localization from three separate experiments. One-way ANOVA with Tukey’s *post hoc* test was used to test for significant difference in percentage of nuclear Flag-tagged protein among the conditions. Statistically significant values are indicated. *, *P < *0.05; ****, *P < *0.0001. n.s., not significant.

Ank13 of O. tsutsugamushi strain Ikeda, a human isolate from Japan that causes severe scrub typhus (41), is 490 amino acids in length (26, 29). It consists of eight tandemly arranged ARs in its N-terminal half, an 86-amino-acid intervening sequence region (ISR) that is unique to Ank13, and a C-terminal F-box that occurs as part of a PRANC (pox proteins repeats of ankyrin - C terminal) domain (Fig. 1A). It is encoded by a single-copy gene (26, 29). Using the National Center for Biotechnology Information (NCBI) Nucleotide Basic Local Alignment Search Tool (BLASTN) (www.blast.ncbi.nlm.nih.gov) with Ikeda *ank13* as the query identified homologs in O. tsutsugamushi Kato strain (Japan), Karp strain isolates UT76 and UT176 (both from northeastern Thailand), and the Wuj/2014 isolate (China), each of which were recovered from patients and for which the complete genomes are in GenBank (42, 43). These homologs exhibited 89 to 92% nucleotide identity, 77 to 81% amino acid identity, and 85 to 87% amino acid similarity with Ikeda Ank13 (see Table S1 in the supplemental material). Primers targeting conserved nucleotides were used to amplify a segment corresponding to Ikeda *ank1*3 nucleotides 853 to 1260 from Karp isolates UT169, UT177, and UT559 (northeastern Thailand), Gilliam strain isolate FPW2016 (western Thailand), and isolates TM2532 (Central Laos), and SV445 (southern Laos) (43–45) (see Fig. S1A), for which genomic sequences are not available in GenBank. Sequencing and aligning the amplicons and their predicated amino acid sequences revealed 88 to 96% nucleotide identity, 79 to 90% amino acid identity, and 85 to 93% amino acid similarity with the corresponding region of Ikeda Ank13 (see Fig. S1B and C). Thus, Ank13 is conserved among phylogenetically and geographically diverse O. tsutsugamushi isolates.

10.1128/mBio.01816-21.1 TABLE S1 Comparison of full-length Ank13 homologs in other O. tsutsugamushi strains or isolates. Download Table S1, DOCX file, 0.01 MB. Copyright © 2021 Adcox et al. 2021 Adcox et al. https://creativecommons.org/licenses/by/4.0/ This content is distributed under the terms of the Creative Commons Attribution 4.0 International license.

10.1128/mBio.01816-21.6 FIG S1 *ank13* is present in the genomes of multiple O. tsutsugamushi isolates. (A) PCR amplification of a conserved *ank13* region among O. tsutsugamushi strains. DNA isolated from mammalian cells infected with the indicated O. tsutsugamushi isolates or a minus template control (–) was subjected to PCR using *ank13*-853F/1260R or universal eubacterial 16S rRNA (U16S) primers followed by agarose gel electrophoresis. Numbers to the left correspond to DNA ladder sizes. The data are representative of three experiments. (B) Alignment of *ank13* sequences. Sequences of each 408-base pair amplicon generated in panel A were aligned with homologous regions for Kato, UT176, and Wuj/2014. (C) *ank13* amplicon sequences in panel B were translated and aligned. (B and C) Indicated numbers correspond to Ikeda. Nucleotides and residues differing from those in Ikeda are denoted by black-shaded white text. Download FIG S1, TIFF file, 3.1 MB. Copyright © 2021 Adcox et al. 2021 Adcox et al. https://creativecommons.org/licenses/by/4.0/ This content is distributed under the terms of the Creative Commons Attribution 4.0 International license.

### Ectopically expressed Ank13 robustly localizes to the nucleus.

HeLa cells are excellent models for studying O. tsutsugamushi-host cell interactions because the bacterium’s infection cycle proceeds comparably in them as in other mammalian cell types and their amenability to transfection allows for correlation between phenotypes observed for infected cells and cells ectopically expressing oriential virulence factors (16, 19, 22, 27, 46–49). To quantify the propensity of Ank13 to localize to the nucleus, HeLa cells transfected to express Flag-Ank13 were examined by indirect immunofluorescence microscopy (Fig. 1B). Subcellular distribution of Flag immunosignal was scored as cytoplasmic, throughout the cell, or nuclear (Fig. 1C; see also Fig. S2). Flag-tagged Ank6, an O. tsutsugamushi effector that cycles in and out of the nucleus to impair NF-κB (16), was a positive control for nuclear localization. Negative controls for nuclear localization were Flag-tagged O. tsutsugamushi Ank9, a Golgi- and ER-tropic effector, and bacterial alkaline phosphatase (BAP), both of which predominantly remain in the cytoplasm but exhibit low level nuclear accumulation when ectopically expressed (16, 27, 29). Flag-Ank13 and Flag-Ank6 exclusively localized to the nucleus in 81 and 40% of transfected cells, respectively, with neither exhibiting strict cytoplasmic distribution (Fig. 1C). Flag-BAP exclusively and Flag-Ank9 near exclusively remained in the cytoplasm. These observations were validated when nuclear and cytoplasmic fractions of HeLa cells expressing the same four proteins were subjected to Western blotting. Screening the fractions with antibodies against the Flag tag, lamin A/C (nuclear fraction loading control), and glyceraldehyde-3-phosphate dehydrogenase (GAPDH; cytoplasmic fraction loading control) confirmed that Flag-Ank13 pronouncedly traffics to the nucleus and does so with a significantly greater efficiency than Flag-Ank6 (Fig. 1D and E).

10.1128/mBio.01816-21.7 FIG S2 Representative images depicting Flag-Ank13 immunosignal localization. Transfected HeLa cells expressing Flag-Ank13 were fixed, probed with Flag antibody, stained with DAPI, and examined by immunofluorescence microscopy. Flag immunosignal localization was scored as being exclusively cytoplasmic (A), throughout the cell (B), or exclusively nuclear (C). Download FIG S2, TIFF file, 1.5 MB. Copyright © 2021 Adcox et al. 2021 Adcox et al. https://creativecommons.org/licenses/by/4.0/ This content is distributed under the terms of the Creative Commons Attribution 4.0 International license.

### *O. tsutsugamushi* temporally upregulates *ank13* expression late during infection.

**FIG 2:**
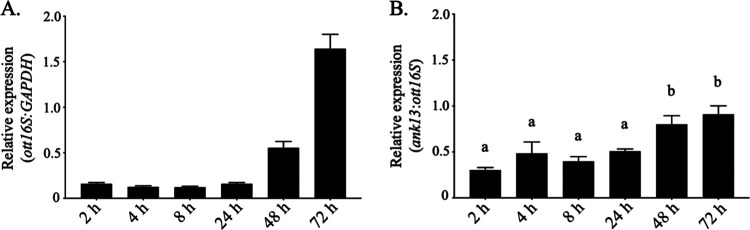
O. tsutsugamushi expresses *ank13* during infection of host cells. HeLa cells were synchronously infected with O. tsutsugamushi, followed by collection of total RNA at the indicated time points. RT-qPCR was performed using gene-specific primers. Relative *O. tsutsugamushi* 16S rRNA gene (*ott16S*)-to-human *GAPDH* (A) and *ank13*-to-*ott16S* expression (B) was determined using the 2^−ΔΔ^*^CT^* method. The data are mean values ± the SD from three experiments performed in triplicate. One-way ANOVA with Tukey’s *post hoc* test was used to test for significant difference of relative *ank13* levels across time points. The mean values indicated by the letter “a” are significantly different from those labeled “b.”

Following entry into host cells, the O. tsutsugamushi population exhibits minimal growth for the first 24 to 48 h but logarithmically expands thereafter until bacteria exit or lyse the cells (46, 47). We previously detected *ank13* transcript by RT-PCR in cells in which the infection had become asynchronous (29). Whether *ank13* expression varies over the course of infection was unknown. Total RNA isolated from synchronously infected HeLa cells at 2, 4, 8, 24, 48, and 72 h was analyzed by RT-qPCR for *ank13* expression normalized to that of O. tsutsugamushi 16S rRNA (*ott16S*). Monitoring *ott16S*-to-*GAPDH* expression supported that, consistent with previous reports (46, 47), the bacterial population did not begin to expand until around 48 h (Fig. 2A). Though *ank13* expression was detected at all time points, significantly higher expression was observed at 48 and 72 h (Fig. 2B), suggesting that O. tsutsugamushi upregulates *ank13* during its expansive replication phase.

### Ank13 is expressed and localizes to the nucleus in *O. tsutsugamushi-*infected cells.

**FIG 3:**
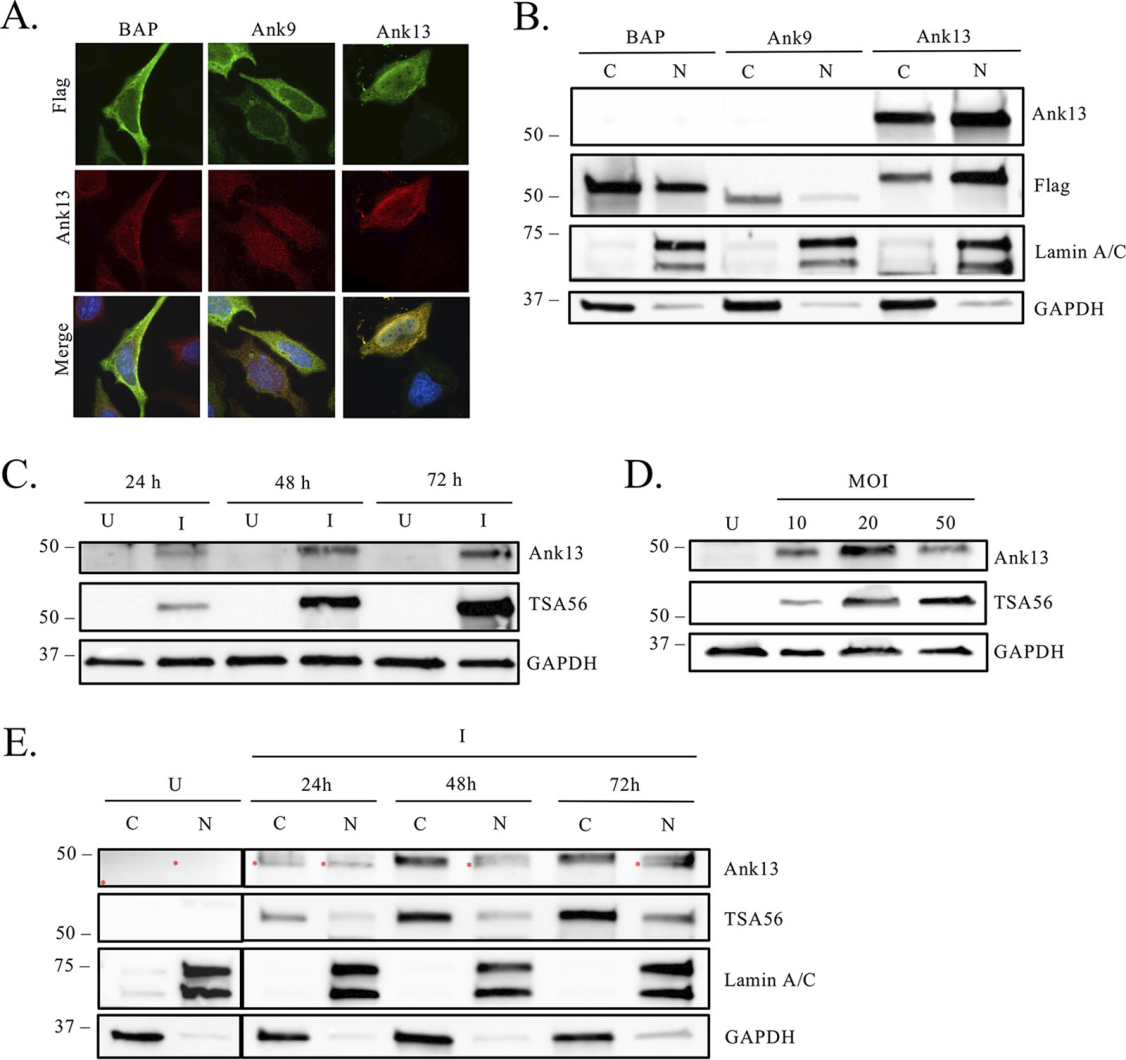
Antiserum specific for Ank13_288-360_ detects ectopically expressed and O. tsutsugamushi Ank13 in infected cells. (A and B) Anti-Ank13_288-360_ (anti-Ank13) specifically recognizes Flag-Ank13. HeLa cells expressing Flag-tagged BAP, Ank9, or Ank13 were either (i) fixed, probed with anti-Flag and anti-Ank13, stained with DAPI, and examined by immunofluorescence microscopy (A) or (ii) lysed and separated into cytoplasmic [C] and nuclear [N] fractions that were subjected to Western blot analyses using lamin A/C, GAPDH, Ank13, and Flag epitope antibodies (B). (C to E) Anti-Ank13 detects bacterium-derived Ank13 in O. tsutsugamushi-infected cells. HeLa cells were either mock [U] or infected [I] with O. tsutsugamushi at an MOI of 10, and whole-cell lysates were collected at 24, 48, and 72 h (C). An MOI of 10, 20, or 50 and whole-cell lysates was collected at 72 h (D), or an MOI of 10 and cytoplasmic [C] and nuclear [N] fractions was collected at 24, 48, and 72 h (E). Western blots were probed with the indicated antibodies. Red asterisks in panel E denote nonspecific host cell-derived bands. The results are representative of at least three experiments with similar results.

To verify that O. tsutsugamushi expresses Ank13 during infection, we generated Ank13-specific antiserum. Through *in silico* analyses, residues 288 to 360 within the ISR were determined to be unique to Ank13 within the Ikeda strain proteome. Rat antiserum specific for this region, here referred to as anti-Ank13, detected Flag-Ank13 in the nuclei and cytoplasm of transfected HeLa cells but did not recognize Flag-tagged BAP or Ank9 (Fig. 3A and B). Anti-Ank13 was then used to screen Western-blotted lysates of uninfected HeLa cells or cells that had been infected with O. tsutsugamushi at a multiplicity of infection (MOI) of 10 for 24, 48, or 72 h or an MOI of 10, 20, or 50 for 72 h (Fig. 3C and D). Infection was verified by screening the blots with antiserum targeting TSA56 (56-kDa type-specific antigen), an abundantly expressed O. tsutsugamushi outer membrane protein (50). Anti-Ank13 recognized a band having an apparent molecular weight slightly below that of the 53.7-kDa size expected for Ank13 in lysates from infected, but not uninfected cells. Anti-Ank13 also detected a comparably sized band in cytosolic and nuclear fractions of infected cells (Fig. 3E). The signal intensity of the Ank13 band increased in both fractions over the 72-h time course, whereas a nonspecifically recognized host cell-derived band did not. Detection of TSA56 at a lower signal intensity in nuclear fractions relative to cytoplasmic fractions from infected cells at each time point is consistent with intranuclear localization of a subpopulation of O. tsutsugamushi organisms (51). Hence, O. tsutsugamushi-expressed Ank13 is present in both the cytoplasm and the nuclei of infected cells.

### Ank13 interacts with the SCF ubiquitin ligase complex in an F-box-dependent manner and its nucleotropism is independent of the classical nuclear import pathway.

**FIG 4:**
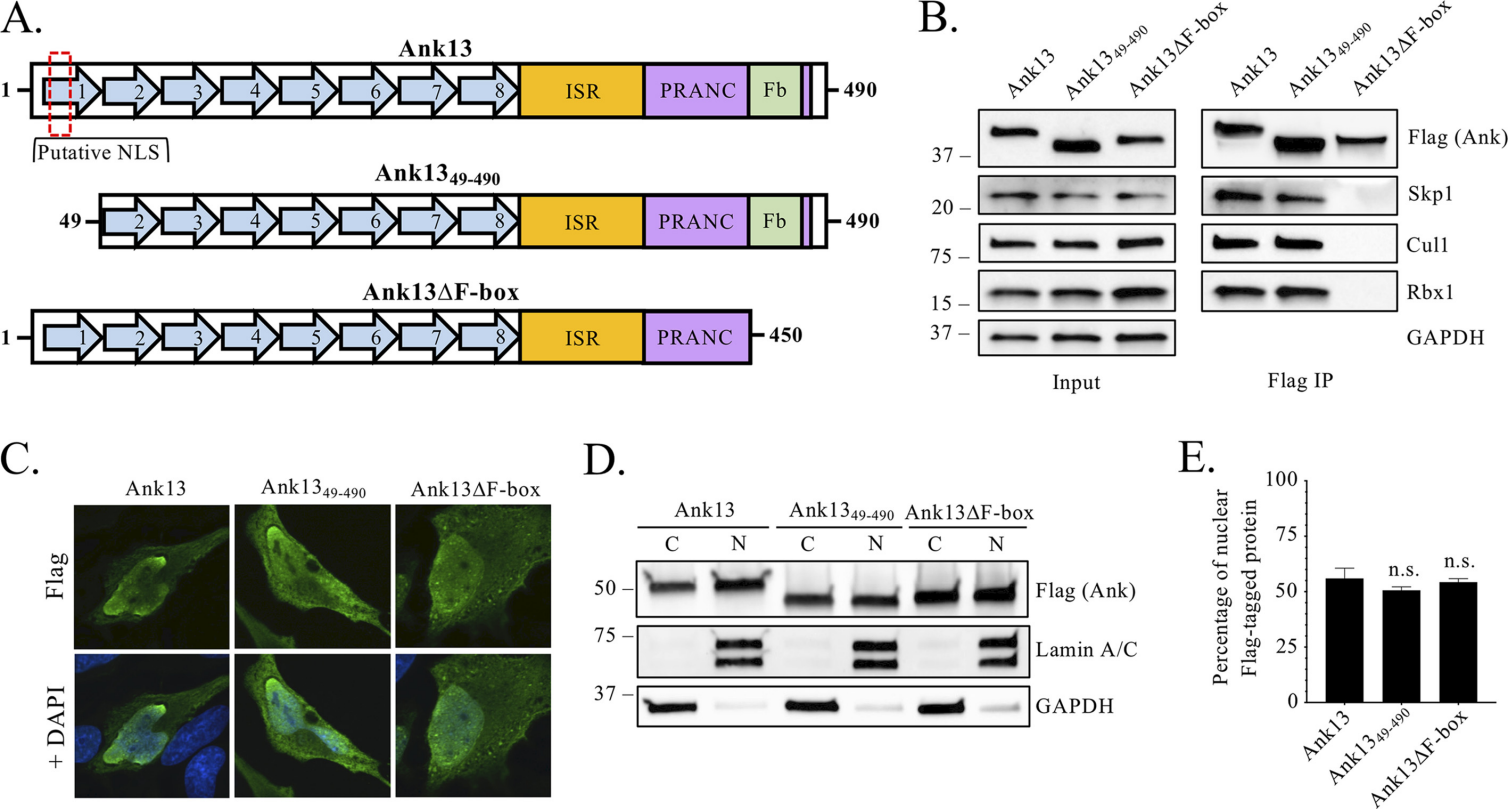
A putative NLS does not contribute to Ank13 nucleotropism. (A) Schematics of wild-type and Ank13 truncated mutant proteins. A red hatched line box indicates the putative NLS that consists of residues 10 to 42 and occurs within ankyrin repeat 1. (B) Confirmation that Ank13ΔF-box fails to interact with SCF ubiquitin ligase components. HeLa cells were transfected to express Flag-tagged Ank13, Ank13_49-490_, or Ank13ΔF-box. Input lysates were subjected to Western blotting with Flag antibody to verify ectopic expression of the proteins of interest; antibodies against Skp1, Cul1, and Rbx1 to confirm their presence; and GAPDH antibody to validate that equivalent amounts of protein were present in each sample. Whole-cell lysates were incubated with Flag antibody-conjugated agarose beads to immunoprecipitate (IP) Flag-tagged proteins and their interacting proteins. The resulting Western blot was probed with indicated antibodies to confirm recovery of the Flag-tagged proteins and assess for Skp1, Cul1, or Rbx1 coimmunoprecipitation. (C to E) Ankyrin repeat 1 (containing the putative NLS) and the F-box of Ank13 are dispensable for nuclear localization. Transfected HeLa cells expressing Flag-Ank13, Flag-Ank13_49-490_, or Flag-Ank13ΔF-box were either fixed, probed with Flag antibody, stained with DAPI, and examined by immunofluorescence microscopy (C) or lysed and resolved into cytoplasmic [C] and nuclear [N] fractions that were subjected to Western blot analyses using the indicated antibodies (D). (E) The nuclear densitometric value was divided by the sum of nuclear and cytoplasmic densitometric values for Flag-tagged proteins in panel D. The quotient was multiplied by 100 to yield the percentage of Flag-tagged protein in the nucleus. Data presented are the percentages (means ± the SD) of Flag-tagged proteins exhibiting nuclear localization from three separate experiments. One-way ANOVA with Tukey’s *post hoc* test was used to assess for significant differences among the conditions. n.s., not significant.

We sought to confirm the ability of Ank13 to interact with SCF ubiquitin ligase machinery and to determine whether it translocates into the nucleus via the classical nuclear import pathway by focusing on the contributions of its C-terminal F-box and putative N-terminal nuclear localization signal (NLS), respectively. cNLS Mapper (52) identified Ank13 residues 10 through 41 as harboring a potential NLS by assigning the region a score of 7 out of a possible 10. Eukaryotic NLS-bearing cargo are delivered into the nucleus by the classical nuclear import pathway, which involves importin α recognition of the NLS, followed by binding of importin β1 to importin α and importin β1-mediated translocation of the ternary complex through the nuclear pore (53). To evaluate the putative NLS, we generated a construct encoding Flag-Ank13_49-490_, which lacks through the end of the first AR (Fig. 4A). This deletion was necessary because the candidate NLS extends to within the first AR. Deleting part of an AR disrupts tertiary structure of an AR domain, but deleting an entire AR does not (54). The Ank13 F-box consists of residues 451 to 471 (Fig. 1A). We previously demonstrated that Flag-Ank13 but not Flag-Ank13_Δ451-490_, here simply referred to as Flag-Ank13ΔF-box (Fig. 4A), precipitated glutathione *S*-transferase-tagged Skp1 (30). Whether the Ank13 F-box is capable of interacting with endogenous Skp1 or any other SCF component was unknown. HeLa cells were transfected to express Flag-tagged Ank13, Ank13_49-490_, or Ank13ΔF-box. Lysates were collected and incubated with Flag antibody-coated beads to immunoprecipitate the Flag-tagged proteins and their interacting partners. Eluted complexes were Western blotted and screened with antibodies for Skp1, Cul1, and Rbx1. Flag-Ank13 and Flag-Ank13_49-490_, but not Flag-Ank13ΔF-box, coprecipitated all three (Fig. 4B), thereby confirming that Ank13 interacts with the endogenous SCF complex in an F-box-dependent manner. Subcellular distribution of Flag immunosignal, as analyzed by both indirect immunofluorescence and Western blotting, demonstrated that Flag-Ank13, Flag-Ank13_49-490_, and Flag-Ank13ΔF-box exhibited comparable accumulation in the nucleus (Fig. 4C to E). Hence, the Ank13 nucleotropism requires neither Ank13 residues 1 through 48 nor its F-box. Moreover, Ank13 residues 10 through 41 do not contain an NLS.

**FIG 5:**
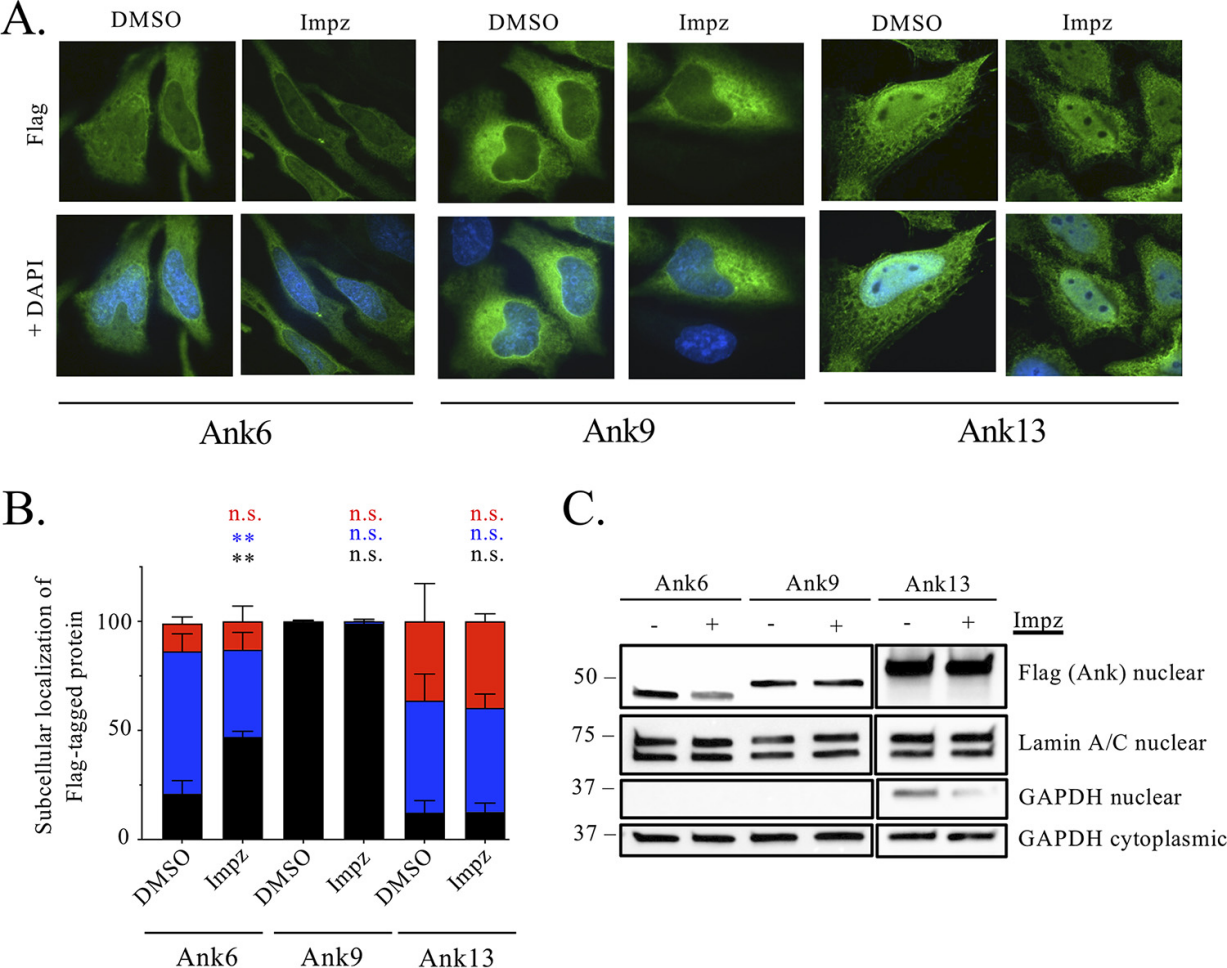
Ank13 nuclear accumulation is importazole-insensitive. HeLa cells expressing Flag-tagged Ank6, Ank9, or Ank13 were treated with importazole (Impz) or DMSO for 3 h and then fixed, probed with Flag antibody, stained with DAPI, and examined by immunofluorescence microscopy. (A) Representative immunofluorescent images. (B) One hundred cells per condition were examined in triplicate to quantify the percentages (means ± the SD) of cells exhibiting Flag immunosignal subcellular localization, scored as being exclusively cytoplasmic (black), throughout the cell (blue), or exclusively in the nucleus (red). Two-way ANOVA with Dunnett’s correction was used to determine significance between subcellular locations of Flag-Ank13 in cells treated with Impz versus DMSO. (C) Transfected HeLa cells were separated into cytoplasmic [C] and nuclear [N] fractions that were subjected to Western blot analyses using Flag antibody to verify Flag-tagged protein expression and antibodies against lamin A/C and GAPDH to confirm fraction purity. The data are representative of three experiments with similar results. Statistically significant values are indicated. **, *P < *0.01; n.s., not significant.

To further confirm that Ank13 translocation into the nucleus occurs independently of classical nuclear import, HeLa cells expressing Flag-tagged Ank13, Ank6, or Ank9 were treated with importazole, a small molecular inhibitor of importin β1 (55), or DMSO vehicle followed by assessment of Flag immunosignal accumulation in the nucleus. As previously reported (16), importazole inhibited Ank6 nuclear translocation but had no effect on Ank9. Flag-Ank13 subcellular localization was comparable between both conditions (Fig. 5A and B). As an additional control to verify the efficacy of importazole treatment, its effect on nuclear import of NLRC5 (NOD-like receptor family CARD domain containing 5), a eukaryotic transcription factor that shuttles into the nucleus by virtue of its NLS (56), was examined. HeLa cells were stimulated with gamma interferon (IFN-γ) to increase NLRC5 expression, followed by importazole treatment and nuclear fractionation. A clear reduction of nuclear NLRC5 in importazole treated cells was observed (see Fig. S3). Finally, transfected HeLa cells expressing Flag-tagged Ank6, Ank9, or Ank13 were treated with importazole, followed by nuclear fractionation and Western blot analyses. Importazole treatment inhibited Ank6 nuclear accumulation but had no effect on the nonspecific low-level nuclear accumulation of Ank9 (Fig. 5C). Ank13 was as abundant in the nuclear fractions of importazole-treated cells as in those of control cells. Overall, Ank13 does not enter the nucleus via the classical nuclear import pathway.

10.1128/mBio.01816-21.8 FIG S3 Verification that importazole prevents importin β1-dependent nuclear trafficking. HeLa cells were treated with IFN-γ for 18 h, followed by the addition of importazole (Impz; +) or DMSO (–) for 3 h prior to being lysed. Cytoplasmic [C] and nuclear [N] fractions were subjected to Western blot analyses using antibody to detect NLRC5, the IFN-γ-dependent nuclear localization of which is importazole-sensitive, and antibodies against lamin A/C and GAPDH to confirm fraction purity. Download FIG S3, TIFF file, 0.1 MB. Copyright © 2021 Adcox et al. 2021 Adcox et al. https://creativecommons.org/licenses/by/4.0/ This content is distributed under the terms of the Creative Commons Attribution 4.0 International license.

### RaDAR-mediated nuclear import of Ank13 requires isoleucine 127.

**FIG 6:**
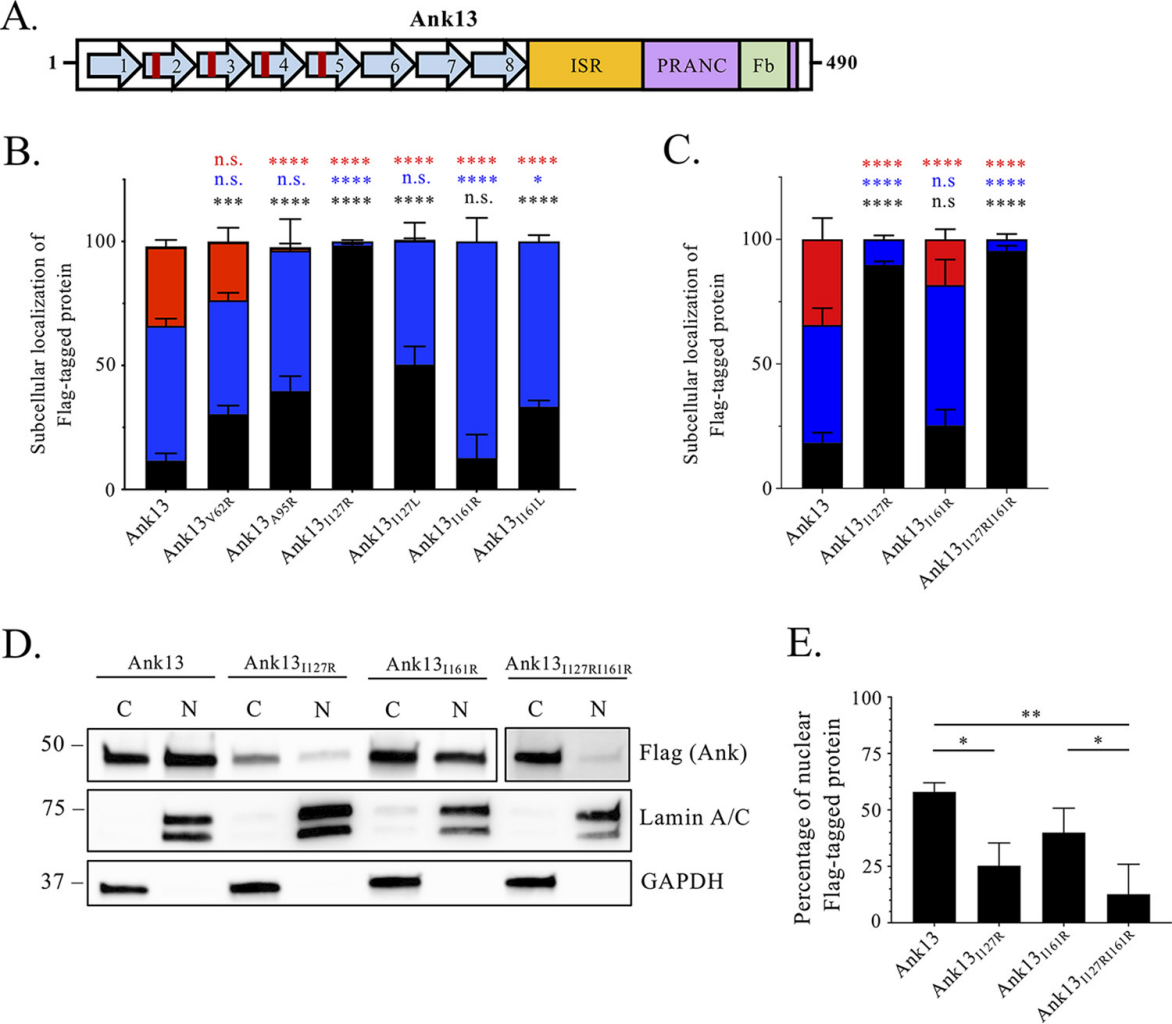
Iso127 and Iso161 are critical for and contribute to Ank13 nuclear localization. (A) Schematic of Ank13 with the relative positions of V62, A95, I127, and I161 denoted as red lines. These amino acids were replaced with R or L to generate indicated mutants. (B to E) Transfected HeLa cells expressing Flag-Ank13 or Flag-Ank13 bearing the indicated amino acid substitutions were either examined by immunofluorescence microscopy (B and C) or Western blot analysis of nuclear [N] and cytoplasmic [C] fractions (D and E). (B and C) One hundred cells were examined per condition in triplicate to quantify the percentages (means ± the SD) of cells exhibiting Flag immunosignal localization, which was scored as being exclusively cytoplasmic (black), throughout the cell (blue), or exclusively in the nucleus (red). Two-way ANOVA with Dunnett’s correction determined significance between subcellular locations of Flag-tagged proteins compared to Flag-Ank13. (D) Western-blotted cytoplasmic [C] and nuclear [N] fractions were probed with lamin A/C and GAPDH antibody to verify fraction purity and Flag antibody to determine localization of Flag-tagged protein. (E) The densitometric value of each Flag-tagged protein in the nucleus was divided by the sum of the densitometric values for nuclear and cytoplasmic signals in panel D. The quotient was multiplied by 100 to yield the percentage of Flag-tagged protein in the nucleus. Data presented are the means ± the SD percentage of Flag-tagged proteins exhibiting nuclear localization from three separate experiments. One-way ANOVA with Tukey’s *post hoc* test was used to assess for significant differences among the conditions. *, *P < *0.05; **, *P < *0.01; ***, *P < *0.001; ****, *P < *0.0001. n.s., not significant.

The RaDAR pathway is an importin-independent nuclear import route by which eukaryotic AR-containing proteins bind RanGDP to be delivered into the nucleus. RanGDP binding requires that one or two, often consecutive, ARs contain a hydrophobic residue—preferentially L, I, or F—or C at the 13th position (57). Ank13 ARs two through five have the following hydrophobic residues at the thirteenth position: AR2 V62, AR3 A95, AR4 I127, and AR5 I161 (Fig. 6A). The study that discovered RaDAR also found that substituting the key hydrophobic AR residues with hydrophilic R strongly disrupted nuclear translocation, while swapping in L or I was less disruptive (57). Flag-Ank13_I127R_ delivery into the nucleus was all but abolished and Flag-Ank13_I127L_ nuclear translocation was lowered by approximately 40% relative to Flag-Ank13 (Fig. 6B). By comparison, Flag-tagged Ank13_V62R_, Ank13_A95R_, and Ank13_I161R_ exhibited more modest reductions in nuclear import efficiency. Due to the essentiality of AR4 I127 for Ank13 nuclear import, because efficient RaDAR-mediated nuclear import of eukaryotic AR-containing transcription factors is reliant on hydrophobic residues at the 13th position in two consecutive ARs (57), and because said 13th positions tend to be more commonly occupied by I than A (57), we examined the nuclear translocation efficiency of Flag-Ank13_I127RI161R_. Though the nuclear translocation defect of Flag-Ank13_I127RI161R_ was slightly stronger than for Flag-Ank13_I127R_, the difference was statistically insignificant (Fig. 6C to E). This was most likely due to the overwhelming inhibitory effect of the I127R versus I161R mutation. Thus, Ank13 AR4 I127 and AR5 I161 are critical and contributory, respectively, for its ability to coopt the RaDAR pathway for transport into the nucleus.

### The ability of Ank13 to interfere with yeast growth requires nuclear translocation and the F-box domain.

**FIG 7:**
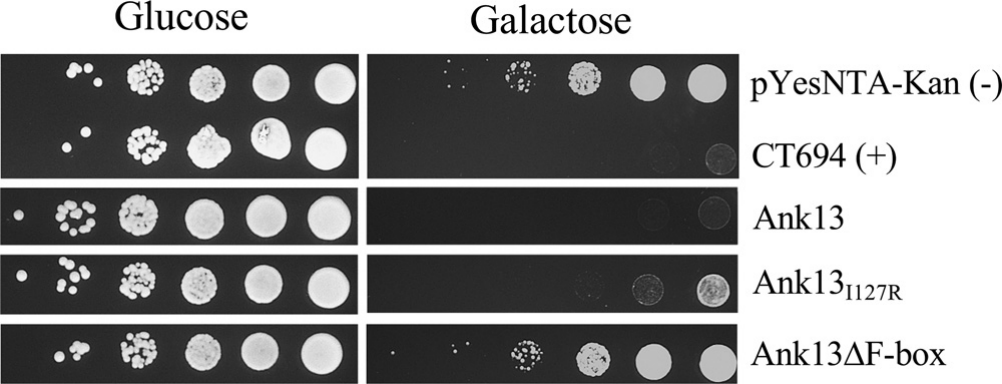
Ank13 interferes with yeast growth in a nuclear translocation- and F-box-dependent manner. S. cerevisiae W303 was transformed with pYesNTA-Kan constructs for expressing C. trachomatis CT694, Ank13, Ank13_I127R_, Ank13ΔF-box, or vector alone. Transformants were diluted to an optical density at 600 nm of 0.2 and spotted as 10-fold serial dilutions onto dropout media containing 2% glucose (noninducing conditions) or 2% galactose (inducing conditions). The data are representative of two to four experiments with similar results.

Toxicity screens using Saccharomyces cerevisiae as a model are useful for studying bacterial effectors due to the high degree of conservation of cellular processes between yeast and mammalian cells. To determine whether Ank13 is toxic to yeast and, if so, whether its ability to translocate into the nucleus and its F-box contribute to its toxicity, Ank13, Ank13_I127R_, and Ank13ΔF-box were inserted into the galactose-inducible vector pYesNTA2. S. cerevisiae W303 transformed with these constructs was grown in uracil dropout media, serially diluted 10-fold, and spotted onto dropout agar containing 2% glucose or 2% galactose. Yeast carrying empty vector or the toxic Chlamydia trachomatis effector CT694 served as negative and positive controls, respectively. Ank13 interfered with yeast growth as severely as CT694 (Fig. 7). Toxicity was reduced in yeast expressing Ank13_I127R_ and abrogated in yeast expressing Ank13ΔF-box. Thus, Ank13 modulates critical eukaryotic biological processes via a mechanism that involves its nucleotropism and requires its ability to interact with host cell ubiquitin ligase components.

### Ank13 alteration of host cell gene expression is nuclear trafficking dependent, F-box dependent, and F-box independent.

**FIG 8:**
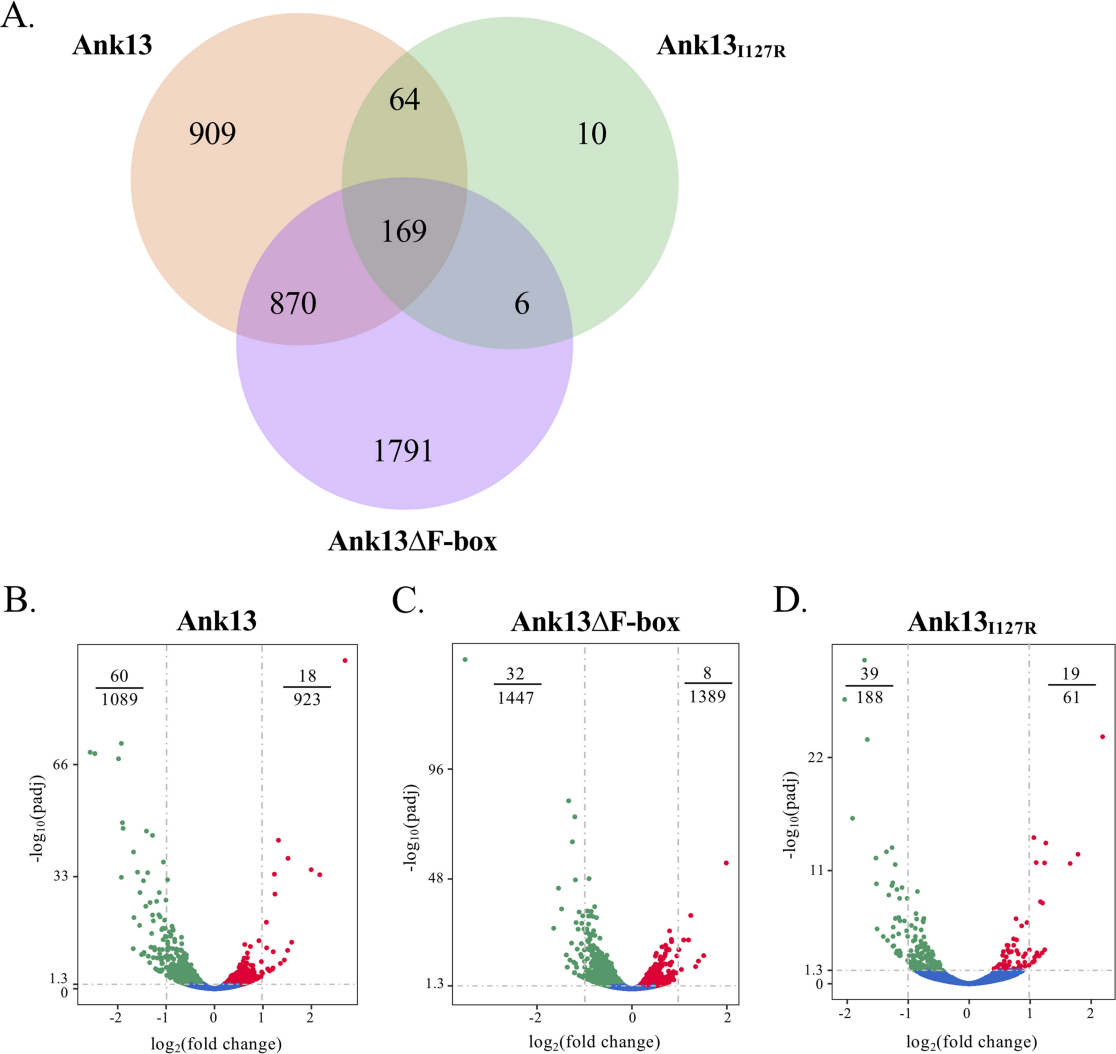
Differential gene expression profiles influenced by Ank13, Ank13_I127R_, or Ank13ΔF-box. (A) Venn diagram showing unique and shared genes differentially expressed by Ank13 (orange, 2012 total), Ank13ΔF-box (purple, 2836 total), or Ank13_I127R_ (green, 249 total). The sum within each circle is the total number of differentially expressed genes in this group and overlapping regions show the number of common genes among comparison groups. (B to D) Volcano plots showing differential expression profiles for cells expressing GFP-tagged Ank13 (B), Ank13ΔF-box (C), or Ank13_I127R_ (D) compared to GFP. Gray horizontal dashed line indicates the threshold for significantly differentially expressed genes (*P*_adj_ < 0.05). Vertical dashes indicate genes exhibiting a log_2_(fold change) of >1 or <−1. Each dot corresponds to an individual gene. Blue dots indicate no significant difference in expression for cells expressing GFP fusions compared to cells expressing GFP. Red and green dots indicate genes that are up- or downregulated, respectively, in cells expressing GFP-fusions versus cells expressing GFP. Fractions in top corners denote the number of genes with log_2_(fold change) of >1 or <−1 out of the total number of differentially expressed genes.

Tools for genetically manipulating O. tsutsugamushi are lacking. As an alternative approach to determine whether Ank13 dysregulates host cell gene expression and assess whether such ability involves its nucleotropism and/or F-box domain, transfected HeLa cells expressing GFP-Ank13, GFP-Ank13_I127R_, GFP-Ank13ΔF-box, or GFP were sorted based on GFP positivity (see Fig. S4) followed by total RNA isolation and RNAseq. Differential gene expression in cells expressing GFP-tagged Ank13, Ank13_I127R_, or Ank13ΔF-box versus GFP was calculated as log_2_(fold change) based on negative binomial distribution (58). Ank13, Ank13ΔF-box, and Ank13_I127R_ promoted differential expression of 2012, 2836, and 249 host genes, respectively (Fig. 8; see also Data Set S1 in the supplemental material). Although Ank13 and Ank13ΔF-box similarly dysregulated 870 genes, the majority of transcriptional changes influenced by each were specific to either protein (Fig. 8A; see also Data Set S1). Only 10 genes were altered in an Ank13_I127R_-specific manner (Fig. 8A; see also Data Set S1). Selecting genes exhibiting at least a 2-fold change in expression among the three transfected populations revealed that the majority were downregulated (Fig. 8B to D; see also Data Set S2). Thus, Ank13 is predominantly a negative regulator of host cell gene expression. While its ability to modulate gene expression primarily relies on its translocation into the nucleus, Ank13 exerts its influence in both F-box-dependent and -independent manners.

10.1128/mBio.01816-21.3 DATA SET S1 These data correlate with Fig. 8A. This sheet lists the differentially expressed genes unique to or shared by each condition. Download Data Set S1, XLSX file, 1 MB. Copyright © 2021 Adcox et al. 2021 Adcox et al. https://creativecommons.org/licenses/by/4.0/ This content is distributed under the terms of the Creative Commons Attribution 4.0 International license.

10.1128/mBio.01816-21.4 DATA SET S2 These data correlate with Fig. 8B, C, and D and Fig. 11. This sheet lists genes down- or upregulated at least 2-fold. Download Data Set S2, XLSX file, 0.6 MB. Copyright © 2021 Adcox et al. 2021 Adcox et al. https://creativecommons.org/licenses/by/4.0/ This content is distributed under the terms of the Creative Commons Attribution 4.0 International license.

10.1128/mBio.01816-21.9 FIG S4 Fluorescence-activated cell sorting of transfected cell populations. Filled grey histograms show control nontransfected HeLa cells. Open histograms indicate transfected HeLa cells expressing GFP (A), GFP-Ank13 (B), GFP-Ank13_I127R_ (C), or GFP-Ank13ΔF-box (D). The horizontal line in each panel demarcates the gate that was set to sort GFP-positive cells. Above each line is indicated the percentages (means ± the SD) of the total number of cells that were sorted for subsequent RNA isolation. Download FIG S4, TIFF file, 0.8 MB. Copyright © 2021 Adcox et al. 2021 Adcox et al. https://creativecommons.org/licenses/by/4.0/ This content is distributed under the terms of the Creative Commons Attribution 4.0 International license.

**FIG 9:**
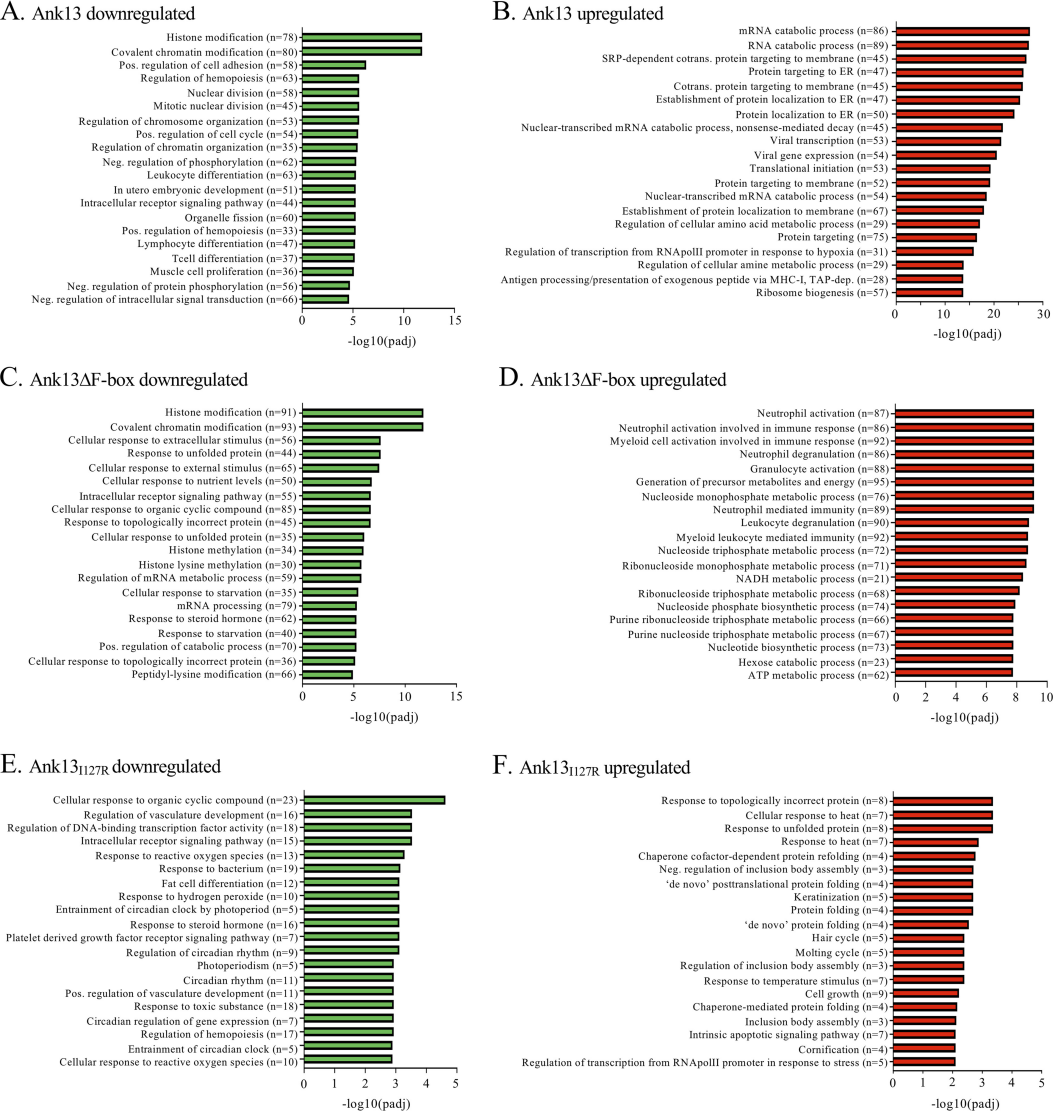
Differentially regulated pathways in cells expressing Ank13, Ank13_I127R_, or Ank13ΔF-box grouped by biological process. Bar plots showing the GO terms subdivided by biological processes that are down- or upregulated in cells expressing GFP-tagged Ank13 (A and B), Ank13ΔF-box (C and D), or Ank13_I127R_ (E and F) compared to cells expressing GFP. Shown are the top 20 most significantly enriched downregulated (green; A, C, and E) and upregulated (red; B, D, and F) GO terms as determined by –log_10_(*P*_adj_) value. *n* = number of genes included in GO term.

Gene ontology (GO) terms were assigned to all differentially regulated genes in cells expressing GFP-Ank13, GFP-Ank13_I127R_, or GFP-Ank13ΔF-box (see Data Set S3). The top 20 GO biological processes up- or downregulated per effector as determined by the –log_10_(*P*_adj_) value are presented in Fig. 9. The two largest categories of genes downregulated by GFP-Ank13 and GFP-Ank13ΔF-box were histone modification and covalent chromatin modification (Fig. 9A and C; see also Fig. S5A and C). Both proteins also downregulated genes associated with histone methylation. All of the remaining top 20 biological processes downregulated by GFP-Ank13ΔF-box were also downregulated by GFP-Ank13 (Fig. 9A and C; see also Data Set S3). Other major categories of genes downregulated by GFP-Ank13 included those associated with regulation of chromatin organization, nuclear division, positive regulation of cell adhesion, and hemopoiesis (Fig. 9A; see also Fig. S5A). From these data it can be concluded that, while Ank13 negatively modulates expression of genes associated with a variety of biological processes, it especially targets those associated with epigenetic functions and does so independently of its F-box.

10.1128/mBio.01816-21.5 DATA SET S3 These data correlate with Fig. 9 and Fig. S5. This sheet lists regulated biological processes (BP GO terms). Top 20 categories within each condition are shaded. Download Data Set S3, XLSX file, 0.8 MB. Copyright © 2021 Adcox et al. 2021 Adcox et al. https://creativecommons.org/licenses/by/4.0/ This content is distributed under the terms of the Creative Commons Attribution 4.0 International license.

10.1128/mBio.01816-21.10 FIG S5 Network analysis of differentially regulated pathways in cells expressing GFP-Ank13 and GFP-Ank13ΔF-box. Concept network plots were used to link genes that are downregulated (A and C) or upregulated (B and D) in cells expressing GFP-Ank13 (A and B) or GFP-Ank13ΔF-box (C and D) versus cells expressing GFP according to their GO terms as defined by biological process. Individual genes are denoted as circles containing gene IDs. The five major GO term nodes (tan) are labeled. Node size positively correlates with the number of associated genes. The scale bar represents the fold change in gene expression. Download FIG S5, TIFF file, 3.6 MB. Copyright © 2021 Adcox et al. 2021 Adcox et al. https://creativecommons.org/licenses/by/4.0/ This content is distributed under the terms of the Creative Commons Attribution 4.0 International license.

The largest two categories of genes upregulated in GFP-Ank13-expressing cells were mRNA and RNA catabolic processes, followed by protein targeting to membranes, especially the endoplasmic reticulum (ER) (Fig. 9B; see also Fig. S5B). Of the top 20 most upregulated GO processes, 12 fell into these and similar categories (Fig. 9B). The top 20 biological processes transcriptionally upregulated by GFP-Ank13ΔF-box were also upregulated by GFP-Ank13 (Fig. 9B and D; see also Fig. S5B and D and Data Set S3). However, whereas GFP-Ank13 upregulated 86 mRNA catabolism genes and 89 RNA catabolism genes (Fig. 9B), GFP-Ank13ΔF-box upregulated only 45 mRNA catabolism genes and no RNA catabolism genes (see Data Set S3). Transcriptional upregulation of ER/membrane-localization processes was also GFP-Ank13 specific. When the up- and downregulated pathways in cells expressing GFP-Ank13_I127R_ were examined, there were markedly fewer differentially expressed genes (Fig. 9E and F). The biological processes themselves were a clear departure from those modulated by GFP-Ank13 and GFP-Ank13ΔF-box, signifying that much of the Ank13 modulatory effect is linked to its nucleotropism (Fig. 9). The most upregulated genes included those associated with topologically incorrect or unfolded proteins, cellular stress, and apoptosis (Fig. 9E and F), suggesting that cytoplasmic accumulation of overexpressed GFP-Ank13_I127R_ invokes cellular stress responses.

Overall, the GO analyses demonstrate that, among the many host cellular processes that Ank13 transcriptionally modulates, it impairs expression of genes involved in epigenetic regulation in an F-box-independent manner and upregulates genes associated with mRNA/RNA catabolism and membrane/ER targeting primarily in an F-box-dependent manner. Both of these phenomena are predicated on the effector’s nucleotropism.

### Genes most strongly downregulated by Ank13 include those involved in transcriptional control and inflammation and correlate with transcriptional trends in *O. tsutsugamushi-*infected cells.

**FIG 10:**
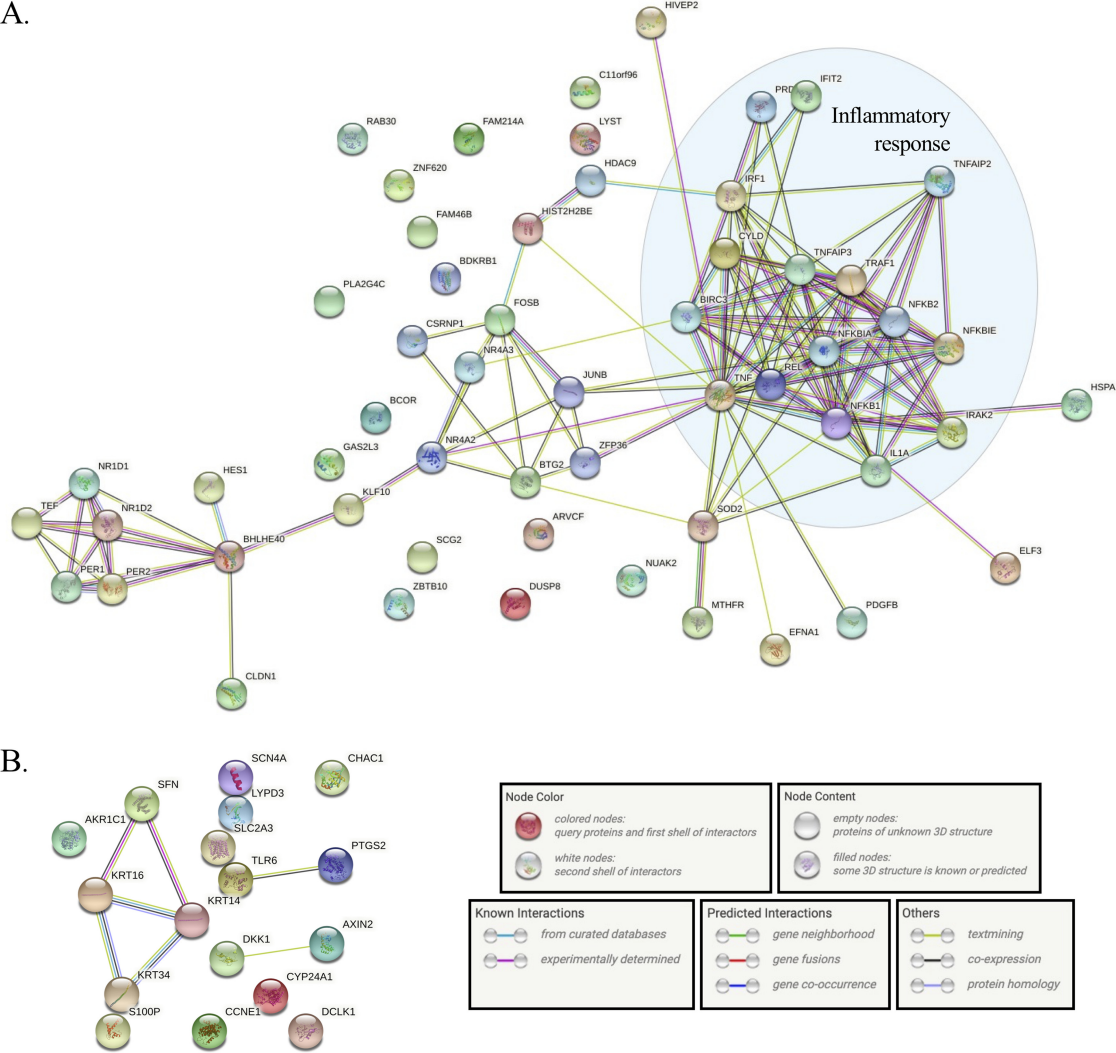
Genes downregulated in cells expressing Ank13 show enriched modulation of the inflammatory response. The 60 host genes that were downregulated (A) or 18 genes that were upregulated (B) ≥2-fold in cells expressing GFP-Ank13 were subjected to STRING analysis. The shaded blue oval in panel A was added manually to denote genes involved in host cell inflammatory response.

Being that Ank13 is primarily a negative transcriptional modulator, we next focused on the 60 genes that were downregulated ≥2-fold in cells expressing GFP-Ank13 (see Data Set S2). We categorized these genes as being downregulated dependent on Ank13 I127 and/or its F-box. Genes downregulated ≥2-fold in cells expressing GFP-Ank13, but neither GFP-Ank13_I127R_ nor GFP-Ank13ΔF-box were interpreted as being inhibited by Ank13 in both nucleotropism- and F-box-dependent manners. Genes meeting this criterion in cells expressing GFP-Ank13 and GFP-Ank13ΔF-box, but not GFP-Ank13_127R_, were considered to be negatively modulated by Ank13 in a nucleotropism-dependent but F-box-independent mechanism. Those downregulated in cells expressing GFP-Ank13 and GFP-Ank13_I127R_, but not GFP-Ank13ΔF-box were regarded as being impaired by Ank13 in an F-box-dependent but nucleotropism-independent manner. Of the 60 genes, 46 were categorizable per these criteria (Table 1). Ank13 downregulates a total of 15 genes involved in transcriptional control in nucleotropism- and F-box-dependent, as well as -independent, fashions. It negatively modulates 15 proinflammatory genes, including NF-κB-related genes in both nucleotropism- and F-box-dependent manners plus interleukin-1α (IL-1α), IL-1 receptor-associated kinase 2, tumor necrosis factor-related genes, and NF-κB inhibitor α via an F-box-dependent mechanism. Supporting this trend, STRING (Search Tool for the Retrieval of Interacting Genes/Proteins) (59) analysis of genes downregulated ≥2-fold by GFP-Ank13 revealed a functional enrichment for the inflammatory response (Fig. 10A). The effector uses its F-box independent of its nucleotropism to downregulate genes involved in cell cycle control, interferon response, mRNA, and histone deacetylase nine. Ank13 also traffics to the nucleus to inhibit transcription of histone cluster and heat shock protein family A genes in an F-box-independent manner. In contrast and as further evidence that Ank13 predominantly acts to transcriptionally downregulate numerous interrelated host cell processes, STRING analysis of genes upregulated ≥2-fold by GFP-Ank13 showed little functional enrichment aside from a few that are involved in keratinization (Fig. 10B).

**TABLE 1:** Host genes downregulated ≥2-fold in cells expressing GFP-Ank13 proteins correlated with their transcriptional profiles in O. tsutsugamushi infected cells^*a*^

Biological group	Gene	Description	Location	Cells expressing GFP-Ank13 proteins			O. tsutsugamushi-infected cells	
				Wild-type log_2_(FC)	I127R log_2_(FC)	ΔF-box log_2_(FC)	4 h log_2_(FC)	48 h log_2_(FC)
Downregulation is nucleotropism and F-box-dependent								
Transcriptional control	ELF3	E74-like factor 3 (Ets domain transcription factor epithelium-specific)	Nucleus	−1.22	NC^*b*^	NC	NC	NC
	BCOR	BCL6 corepressor	Nucleus	−1.02	−0.74	NC	NC	NC
	HES1	Hes family bHLH transcription factor 1	Nucleus	−1.20	−0.67	−0.63	NC	NC
	BHLHE40	Basic helix-loop-helix family member e40	Nucleus	−1.06	−0.48	−0.19	NC	0.64
	PRDM1	PR domain containing 1 with ZNF domain	Nucleus	−1.26	NC	−0.66	NC	1.14
Inflammatory response	BDKRB1	Bradykinin receptor B1	Plasma membrane	−1.46	NC	NC	NC	−1.27
	CYLD	Cylindromatosis (turban tumor syndrome)	Plasma membrane, cytoplasm	−1.06	−0.78	−0.54	NC	NC
	REL	v-rel avian reticuloendotheliosis viral oncogene homolog	Nucleus	−1.46	NC	−0.57	NC	NC
	NFKBIE	Nuclear factor of kappa light polypeptide gene enhancer in B cells inhibitor epsilon	Cytoplasm	−1.10	NC	NC	NC	NC
	NFKB1	Nuclear factor of kappa light polypeptide gene enhancer in B cells 1	Nucleus, cytoplasm	−1.14	−0.86	−0.50	0.41	NC
	NFKB2	Nuclear factor of kappa light polypeptide gene enhancer in B cells 2 (p49/p100)	Nucleus, cytoplasm	−1.41	−0.74	−0.48	0.65	NC
								
Downregulation is F-box dependent								
Transcriptional control	CSRNP1	Cysteine-serine-rich nuclear protein 1	Nucleus	−1.38	−1.27	−0.81	NC	NC
	TEF	Thyrotrophic embryonic factor	Nucleus	−1.18	−1.21	NC	NC	NC
	HIVEP2	Human immunodeficiency virus type I enhancer binding protein 2	Nucleus	−1.28	−0.91	−0.68	NC	NC
	ZFP36	ZFP36 ring finger protein	Nucleus	−1.08	−0.95	−0.79	NC	NC
	KLF10	Kruppel-like factor 10	Nucleus	−1.28	−1.02	−0.68	NC	NC
	ZNF620	Zinc finger protein 620	Nucleus	−1.08	−0.89	NC	NC	NC
	LBX2-AS1	LBX2 antisense RNA 1	Nucleus	−1.08	−1.01	−0.48	NC	NC
Inflammatory response	IL1A	IL-1α	Extracellular	−1.08	−1.01	−0.43	NC	NC
	IRAK2	IL-1 receptor associated kinase 2	Plasma membrane, extracellular, cytoskeleton, nucleus	−1.59	−1.14	NC	1.21	NC
	TNF	Tumor necrosis factor	Plasma membrane, extracellular	−1.34	−1.41	NC	NC	NC
	TRAF1	TNF receptor-associated factor 1	Plasma membrane, cytoplasm	−2.57	−2.04	−0.76	NC	NC
	TNFAIP2	TNF alpha-induced protein 2	Extracellular	−1.47	−1.36	−0.39	NC	NC
	TNFAIP3	TNF alpha induced protein 3	Nucleus, lysosome	−1.98	−1.53	−0.68	NC	NC
	NUAK2	NUAK family SNF1-like kinase 2	Nucleus	−1.30	−1.25	−0.57	NC	NC
	NFKBIA	Nuclear factor of kappa light polypeptide gene enhancer in B cells inhibitor alpha	Nucleus (cytoplasm)	−1.67	−1.03	−0.39	NC	NC
	BIRC3	Baculoviral IAP repeat containing 3	Nucleus	−1.90	−1.16	−0.30	NC	NC
IFN response	IRF1	Interferon regulatory factor 1	Nucleus	−1.54	−1.27	−0.59	NC	NC
	IFIT2	Interferon induced protein with tetratricopeptide repeats 2	Endoplasmic reticulum	−1.12	−0.95	NC	NC	2.49
mRNA	FAM46B	Family with sequence similarity 46 member B	Cytoskeleton, nucleus	−1.10	−1.13	−0.59	−0.63	−1.98
	NOCT	Nocturnin	Cytoskeleton	−0.97	−1.10	−0.62	NC	NC
Histone deacetylase	HDAC9	Histone deacetylase 9	Nucleus	−1.19	−0.92	NC	NC	NC
Cell cycle control	ADIRF-AS1	ADIRF antisense RNA 1	Nucleus	−1.55	−1.25	−0.87	NC	NC
	NR1D2	Nuclear receptor subfamily 1 group D member 2	Nucleus	−1.12	−1.16	−0.50	NC	NC
	FAM214A	Family with sequence similarity 214 member A	Nucleus	−1.24	−0.93	−0.74	NC	NC
	PER2	Period circadian clock 2	Nucleus	−1.24	−0.96	NC	NC	NC
	PER1	Period circadian clock 1	Nucleus	−1.92	−1.72	−0.83	NC	0.52
	NR1D1	Nuclear receptor subfamily 1 group D member 1	Nucleus	−1.92	−1.91	−0.43	NC	0.69
								
Downregulation is nucleotropism-dependent								
Transcriptional control	DUSP8	Dual specificity phosphatase 8	Nucleus	−1.09	−0.71	−1.13	NC	−1.12
	NR4A2	Nuclear receptor subfamily 4 group A member 2	Nucleus	−1.15	−0.77	−0.95	−0.57	NC
	C11orf96	Chromosome 11 open reading frame 96	NC	−1.14	NC	−0.94	NC	1.94
Heat shock proteins	HSPA1A	Heat shock protein family A (Hsp70) member 1A	Cytoskeleton, nucleus	−0.66	1.20	−1.11	NC	−0.63
	HSPA1B	Heat shock protein family A (Hsp70) member 1B	Cytoskeleton	−0.86	1.06	−1.19	NC	NC
	HSPA6	Heat shock protein family A (Hsp70) member 6	Plasma membrane, cytoskeleton, nucleus, extracellular	−2.47	0.81	−3.51	NC	NC
Histone cluster	HIST1H1C	Histone cluster 1 H1c	Nucleus	−0.73	NC	−1.27	NC	NC
	HIST1H1E	Histone cluster 1 H1e	Nucleus	−0.57	NC	−1.11	NC	NC

a FC, fold change.

b NC, no change. This refers to genes that exhibited no statistically significant change in expression between cells expressing a given GFP-Ank13 protein versus GFP or infected cells versus uninfected cells.

**FIG 11:**
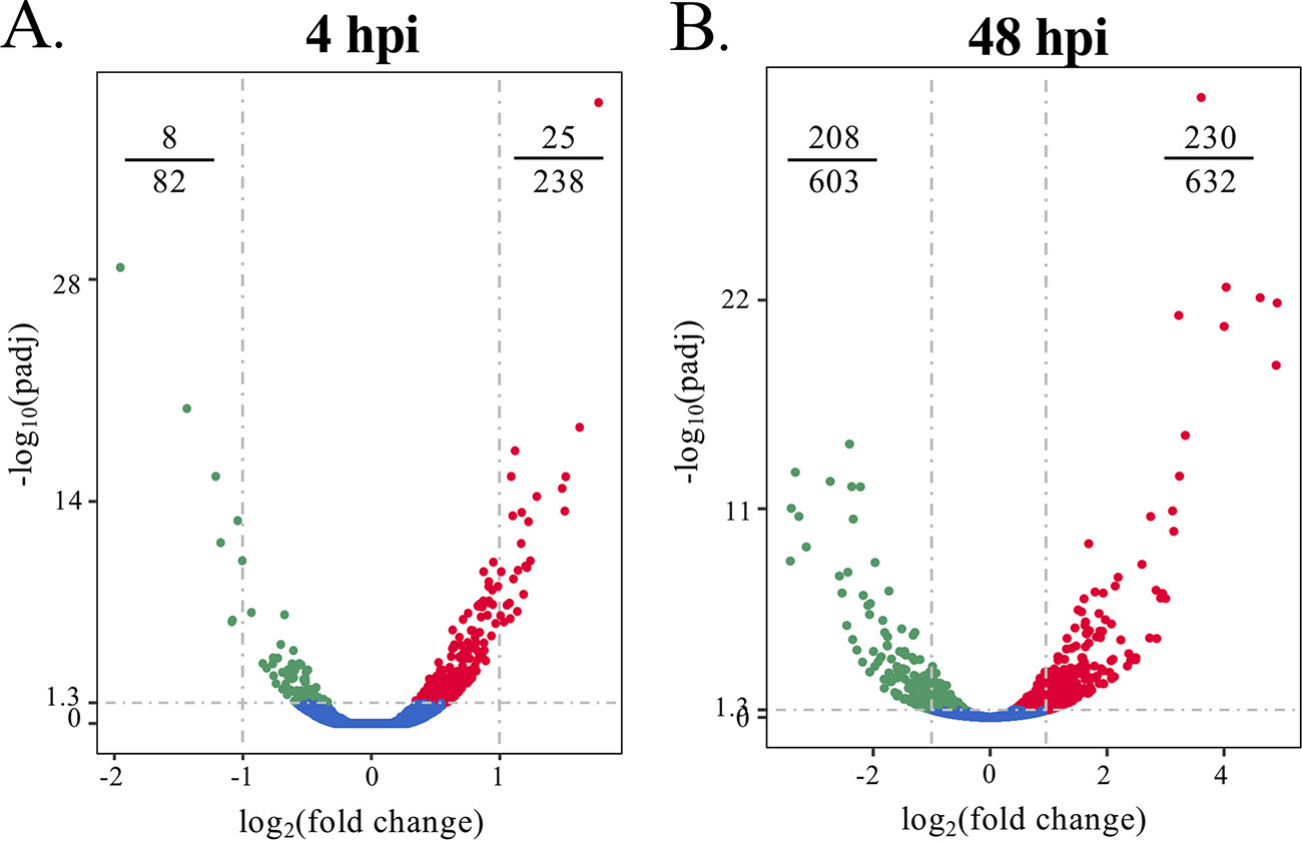
Differential gene expression profiles influenced by O. tsutsugamushi infection. Volcano plots showing differential expression profiles for cells infected with O. tsutsugamushi for 4 h (A) or 48 h (B) compared to uninfected cells. Gray horizontal dashed line indicates the threshold for significantly differentially expressed genes (*P*_adj_ < 0.05). Vertical dashes indicate genes exhibiting a log_2_(fold change) of >1 or <−1. Each dot corresponds to an individual gene. Blue dots indicate no significant difference in infected cells compared to uninfected cells. Red and green dots indicate genes that are up- or downregulated, respectively, in cells expressing GFP fusions versus cells expressing GFP. Fractions in top corners denote the number of genes with log_2_(fold change) of >1 or <−1 out of the total number of differentially expressed genes.

To assess for correlations between expression profiles induced by GFP-Ank13 and O. tsutsugamushi infection, RNA-seq was performed on HeLa cells that had been infected for 4 or 48 h, the latter time point corresponding to when Ank13 is detectable by Western blotting. Host gene expression in infected cells was measured relative to uninfected controls. There was an ∼4-fold increase in the number of differentially expressed genes at 48 h versus 4 h (Fig. 11; see also Data Set S2). Included in the top 20 GO biological processes upregulated at 4 or 48 h were genes involved in recognition of and response to pathogens, cell proliferation, positive regulation of the mitogen-activated protein kinase cascade, and negative regulation of protein phosphorylation (see Data Set S3). Most of the genes downregulated at 4 h were associated with cell differentiation (see Data Set S3). Consistent with O. tsutsugamushi countering immune processes, genes downregulated at 48 h included those involved in leukocyte activation, antigen processing, and antigen presentation (see Data Set S3). The most striking observation was that of the 46 genes that GFP-Ank13 inhibits ≥2-fold, 40 were either not expressed at both infection time points or were more downregulated at 48 h than 4 h (Table 1). Indeed, aligned with prior reports that O. tsutsugamushi stimulates the NF-κB response initially following invasion but then actively represses it as infection proceeds (16, 20), NFKB1 and NFKB2 were among several genes upregulated at 4 h but downregulated at 48 h. Overall, the majority of the genes that GFP-Ank13 most strongly represses, which include those involved in transcriptional regulation and the inflammatory response, are either quiescent or downregulated in O. tsutsugamushi infected cells at a time point when the bacterium expresses Ank13. We conclude that Ank13 contributes to the pathogen’s ability to transcriptionally modulate host cell responses during infection.

## DISCUSSION

Intracellular bacteria use secreted effectors that coopt, subvert, and manipulate eukaryotic processes to generate niches inside host cells. The expansive role of nucleomodulins in this approach has only recently begun to be realized. By targeting host cells at the transcriptional level, nucleomodulins have the capacity to globally alter any number of cellular responses to facilitate microbial colonization (1, 2). This strategy is envisioned to be especially useful for intracytoplasmic microbes, such as O. tsutsugamushi, which are prone to detection by pathogen recognition receptors that invoke potent innate antimicrobial responses and activate neighboring cells to mount adaptive immunity (60). Indeed, although O. tsutsugamushi initially sets off antimicrobial pathways upon host cell invasion, the bacterium ultimately quells these responses and sustains this suppression for the remainder of infection even as it replicates to high numbers in the cytoplasm (16, 18–23). This study established Ank13 as the first *bona fide* O. tsutsugamushi nucleomodulin and one that transcriptionally dysregulates multiple host pathways, including the inflammatory response.

Ank13 is also the first microbial protein identified to exploit the RaDAR pathway for nuclear entry. Eukaryotic RaDAR substrates classically have an I, L, F, or C at the 13th position of an AR as the primary point of interaction required for nuclear import and a second such residue in another, often adjacent AR that is contributory but not essential (57). Consistent with this trend, Ank13 I127 of AR4 is indispensable for and I161 of AR5 minorly aids the effector’s nucleotropism. Other nucleomodulins potentially exploit RaDAR. AnkA, a nucleomodulin of the *Rickettsiales* pathogen Anaplasma phagocytophilum has conspicuous hydrophobic residues at the thirteenth position of consecutive ARs (61). O. tsutsugamushi Ank1 and Ank6 are putative nucleomodulins because they inhibit NF-κB nuclear accumulation and its ability to activate transcription of a fluorescence reporter but have yet to be shown to directly alter expression of host genes. Ank1 and Ank6 utilize the importin α/β1-dependent canonical nuclear localization route (16). Therefore, different O. tsutsugamushi effectors coopt two distinct eukaryotic nuclear import pathways. Functional diversification of O. tsutsugamushi Anks is also reflected in their spatiotemporal expression. Ank13 is most abundantly expressed and detectable in the nucleus during bacterial log-phase growth when risk for pathogen recognition by the host cell is highest. This period coincides with when the effector’s role in transcriptionally countering antimicrobial and other responses would be most needed. In contrast, expression of *ank4*, which contributes to invoking ER stress, coincides with induction of the unfolded protein response that provides amino acids to fuel O. tsutsugamushi growth (46).

Ank13 is predominantly a negative regulator of host cell gene expression. Its ability to alter host transcription involves its nucleotropism and interaction with the SCF ubiquitin ligase complex in both synergistic and mutually exclusive manners. Ank13_I127R_, which is incapable of nuclear translocation but retains the F-box, dysregulates expression of ∼10-fold fewer genes than nucleotropic Ank13 and Ank13ΔF-box. Thus, Ank13 primarily targets host cell transcription within the nucleus but also does so in the cytoplasm conceivably by interacting with host cell transcription factors via its AR domain and promoting their polyubiquitination and proteasomal degradation in an F-box-dependent fashion. This phenomenon is reflected by the presence of O. tsutsugamushi Ank13 and GFP-Ank13 in both the nucleus and cytoplasm. Histone modification is one of the most prominent processes targeted by bacterial effectors for epigenetic control (62). Ank13 and Ank13ΔF-box commonly target numerous biological processes with histone modification, histone methylation, and chromatin modification being key among them. This means that Ank13 acts in the nucleus by negatively regulating cohorts of genes involved in large scale chromosome remodeling, potentially enacting epigenetic reprogramming of the host transcriptome and does so in an F-box-independent fashion. Perhaps Ank13 accomplishes this task by binding directly to distinct chromosomal regions, as has been demonstrated for AnkA and nucleomodulins Ank200, TRP32, TRP47, and TRP120 of another *Rickettsiales* member, Ehrlichia chaffeensis (61, 63–68). Alternatively, Ank13 could interact with transcription factors to sterically hinder their access to DNA.

The ubiquitin-proteasome system regulates transcription via polyubiquitination that leads to removal of DNA-associated proteins (69, 70). Cul1, an Ank13 interacting partner and SCF component, is abundant in the nucleus and associates with 23% of all DNA-associated protein degradation sites (69). In addition to commandeering the SCF complex in the cytoplasm, Ank13 does so in the nucleus as its major means of modulating gene expression. Indeed, Ank13 downregulates the most host genes by functioning in the nucleus in an F-box-dependent manner, as GFP-Ank13 alters transcription of roughly 2,000 distinct genes relative to GFP-Ank13ΔF-box. The importance of the F-box to the ability of Ank13 to globally modulate eukaryotic processes is underscored by the yeast toxicity screen. Whereas Ank13_I127R_ toxicity was reduced 10-fold versus Ank13, removing the F-box lowered Ank13 toxicity 1,000-fold to render it nontoxic. Hence, Ank13 toxicity is predominantly linked to F-box-dependent modulation—most likely ubiquitination/proteasomal degradation—of host nuclear and cytoplasmic proteins that are critical for yeast survival.

The two largest categories of biological processes that Ank13 upregulates are associated with RNA/mRNA catabolism and protein targeting to membranes, the ER in particular. The former could be due to a cellular response to the global transcriptional repression that the effector induces or could be an Ank13-induced response to promote degradation of microRNAs, which contribute to antimicrobial responses and are known targets of various pathogens (62). The increase in ER-targeting genes correlates with O. tsutsugamushi induction of ER stress, the unfolded protein response, and secretory pathway inhibition (27, 46).

Many of the genes that Ank13 most strongly dysregulates correlate with phenotypes observed for O. tsutsugamushi-infected cells. One of the largest cohorts of most strongly downregulated genes are those involved in the inflammatory response, especially NF-κB. O. tsutsugamushi activates NF-κB translocation into the nucleus during the initial hours of infection, but this is soon reversed and remains so throughout infection (16, 20, 21). Whereas Ank1 and Ank6 impair NF-κB accumulation in the nucleus (16), Ank13 contributes to the repression of this pathway by transcriptionally downregulating NF-κB and related genes in F-box-dependent manners in the nucleus and cytoplasm. In agreement with these data, NFKB1, NFKB2, and several other pathogen recognition-induced genes that are upregulated in O. tsutsugamushi-infected cells at 4 h are either downregulated or quiescent by 48 h, a time point that coincides with Ank13 expression. Moreover, nearly all of the genes that are most strongly downregulated by GFP-Ank13 are suppressed in infected cells.

In sum, our report identifies O. tsutsugamushi Ank13 as a novel nucleomodulin and the first such effector to coopt RaDAR for nuclear translocation. Ank13 is a multifaceted protein that functions in the nucleus and cytoplasm via F-box-dependent and -independent manners to globally reprogram host cell transcription. Its conservation among scrub typhus patient isolates suggests that it serves an important role in colonizing mammalian hosts. Given the similarities between gene expression profiles of cells infected with O. tsutsugamushi and ectopically expressing Ank13, we posit that the bacterium deploys Ank13 as a key part of a global reprogramming strategy that maintains the host cell environment as a permissive niche.

## MATERIALS AND METHODS

### Cultivation of cell lines and *O. tsutsugamushi* infections.

Uninfected HeLa cells (CCL-2; American Type Culture Collection [ATCC], Manassas, VA) and HeLa cells infected with O. tsutsugamushi str. Ikeda (NC_010793.1) were maintained as previously described (16). To obtain O. tsutsugamushi for experimental use, infected (≥90%) HeLa cells that had been inoculated 72 to 96 h prior were mechanically disrupted using glass beads, followed by differential centrifugation to recover host cell-free bacteria as described previously (16). Unless stated otherwise, synchronous infections were performed using an MOI of 10. Experiments were verified for achieving the targeted MOI by assessing duplicate coverslips using antiserum specific for TSA56 (27) and immunofluorescence microscopy as described below.

### Analysis of *ank13* homologs.

The National Center for Biotechnology Information (NCBI) Nucleotide Basic Local Alignment Search Tool (BLAST; www.blast.ncbi.nlm.nih.gov) was used to identify homologs of Ikeda str. *ank13* (OTT_RS04140) present in the genomes of other O. tsutsugamushi strains in GenBank. Homologs were identified in Kato (KATO_02023), UT76 (UT76HP_00714), UT176 (UT176_01464), and Wuj/2014 (F0363_02935). To identify homologs in isolates for which genomic information was unavailable, primers *ank13*-853F and *ank13*-1260R (see Table S2), which target nucleotides corresponding to Ikeda *ank13* 853 to 1260 and are identical among the Kato, UT76, UT176, and Wuj/2014 *ank13* homologs, were used to amplify DNA from strains FPW2016, TM2532, SV445, UT169, UT177, and UT559 (71). PCR was performed using MyTaq DNA polymerase (Bioline, Taunton, MA). After an initial denaturing step at 95°C for 1 min, thermal cycling conditions were 35 cycles of 95°C for 15 s, 55°C for 15 s, and 72°C for 10 s, followed by a final extension at 72°C for 30 s. Amplicons were analyzed with 2.0% agarose gels in 40 mM Tris-acetate–2 mM EDTA (pH 8). To ensure the integrity of the template DNA and appropriate thermal cycling conditions, reactions were simultaneously conducted using primers that target the conserved eubacterial 16S rRNA sequence (71). Bands were excised and purified using the QIAquickGel Extraction kit (Qiagen, Germantown, MD). Isolated DNA was submitted for Sanger sequencing using *ank13*-853F and *ank13*-1260R primers (Genewiz, South Plainfield, NJ). The resulting sequences were aligned and analyzed using MegAlign, part of the Lasergene 15.3 software package (DNASTAR, Madison, WI). New partial coding sequences for *ank13* homologs have been deposited in GenBank for FPW2016 (MZ338370), TM2532 (MZ338375), SV445 (MZ338374), UT169 (MZ338371), UT177 (MZ338372), and UT559 (MZ338373).

10.1128/mBio.01816-21.2 TABLE S2 Oligonucleotides utilized in this study. Download Table S2, DOCX file, 0.02 MB. Copyright © 2021 Adcox et al. 2021 Adcox et al. https://creativecommons.org/licenses/by/4.0/ This content is distributed under the terms of the Creative Commons Attribution 4.0 International license.

### Plasmid constructs.

pFlag-Ank13 and pGFP-Ank13 were generated previously (29). Constructs encoding N-terminally Flag- and/or GFP-tagged Ank13_49-490_, Ank13ΔF-box, Ank13_V62R_, Ank13_A95R_, Ank13_I127R_, Ank13_I127L_, Ank13_I161R_, and Ank13_I161L_ were generated using the TaKaRa Bio USA (San Francisco, CA) In-Fusion Mutagenesis protocol and pFlag-Ank13 or pGFP-Ank13 as the template. Primers used to introduce mutations (see Table S2) were designed using the In-Fusion Cloning Primer Design Tool v1.0 (TaKaRa Bio). pFlag-Ank13_I127RI161R_ was made according to the same protocol and using pFlag-Ank13_I127R_ as the template. pGFP-Ank13ΔF-box was constructed by digesting pFlag-Ank13ΔF-box with EcoRI and BamHI and subcloning the released restriction fragment encoding Ank13ΔF-box into the multicloning site of pEGFP-C1 (29). All plasmid constructs were confirmed by sequence analysis (Genewiz). pFlag-BAP (Sigma-Aldrich, St. Louis, MO), pFlag-Ank6 (29), pFlag-Ank9 (29), and empty pEGFP-C1 (29) were included as controls for experiments involving Flag- or GFP-tagged Ank13.

### Ank13 antiserum generation.

NCBI Protein BLAST (www.blast.ncbi.nlm.nih.gov) was used to confirm that Ank13 (WP_012461452.1) residues 288 to 360, encoded by *ank13* nucleotides 862 to 1080, are unique within the annotated O. tsutsugamushi Ikeda str. proteome. An Escherichia coli codon-optimized DNA sequence consisting of two tryptophan codons, followed by two successive repeats of *ank13* nucleotides 862 to 1080, was synthesized and cloned into pET-45b(+) downstream and in-frame with a 6×His tag coding sequence by GenScript (Piscataway, NJ). The two tryptophans were included to facilitate accurate protein concentration determination. The resulting construct was propagated in E. coli Stellar Competent Cells (TaKaRa Bio), miniprepped, and transformed into E. coli BL21(DE3) cells (MilliporeSigma, Burlington, MA). After induction with 1 mM IPTG (isopropyl-β-d-thiogalactopyranoside), E. coli was lysed and the 6×His-tagged chimeric Ank13_288-360_-Ank13_288-360_ protein was purified from the insoluble phase as described previously (72). Briefly, cAnk13 was purified under nondenaturing conditions via gravity flow utilizing nickel-charged Poly-Prep Chromatography Columns (Bio-Rad, Hercules, CA) and 8 M urea buffer, followed by determination of protein concentration using a bicinchoninic acid assay (Thermo Fisher Scientific, Waltham, MA). An 8-week-old female Sprague-Dawley rat was immunized with 50 μg of cAnk13 emulsified in a 1:1 ratio with complete Freund adjuvant and administered in a total volume of 400 μl. At weeks 3 and 5, the rat was boosted with 25 μg of protein in incomplete Freund adjuvant. At week 6, the rat was euthanized by CO_2_ asphyxiation, blood was collected by cardiac puncture, and the Ank13 antiserum recovered. All animal research was performed under the approval the Institutional Animal Care and Use Committee at Virginia Commonwealth University (protocol AD10000387).

### Transfection.

HeLa cells grown to approximately 90% confluence were transfected with plasmid DNA using Lipofectamine 2000 (Invitrogen, Carlsbad, CA) and incubated at 37°C in a humidified incubator at 5% atmospheric CO_2_ for 18 to 24 h. The amount of plasmid DNA used for transfections was modified from that recommended by the Lipofectamine 2000 protocol (Invitrogen) per plasmid to accommodate various levels of transfection efficiency, as determined by enumerating Flag immunosignal-positive cells in indirect immunofluorescence assays or according to Western blot densitometric signal. Spent medium was removed. The cells were washed once with phosphate-buffered saline (PBS; 1.05 mM KH_2_PO_4_, 155 mM NaCl, 2.96 mM Na_2_HPO_4_ [pH 7.4]) before being processed for immunofluorescence microscopy, Western blotting, immunoprecipitation, or cell sorting.

### Immunofluorescence microscopy.

HeLa cells were seeded onto glass coverslips within 24-well plates and transfected to express Flag-tagged protein for 18 h. To inhibit nuclear import, 3 h prior to collection (16 h posttransfection), spent medium was replaced with fresh media containing either 50 μM importazole (Sigma-Aldrich) or dimethyl sulfoxide (DMSO) as a vehicle control prior to fixation. Fixed cells were washed with PBS prior to fixation and permeabilization with −20°C methanol. Coverslips were blocked in 5% (vol/vol) bovine serum albumin (BSA) in PBS. Coverslips were then incubated with rabbit or mouse anti-Flag (Sigma-Aldrich [F1804], 1:1000) or rat anti-Ank13 (1:1,000), followed by incubation with Alexa Fluor 488-conjugated goat anti-rabbit or -mouse and/or Alexa Fluor 594-conjugated goat anti-rat (Invitrogen, 1:1,000) in 5% BSA. Blocking and antibody incubations were performed for 1 h at room temperature with three PBS washes between each step. Samples were incubated with 0.1 μg ml^−1^ DAPI (4′,6′-diamidino-2-phenylindole; Invitrogen) in PBS for 1 min, washed three times with PBS, and mounted with ProLong Gold Antifade mounting media (Invitrogen). Coverslips were imaged with an Olympus BX51 spinning disc confocal microscope (Olympus, Shinjuku City, Tokyo, Japan). Cells were scored for immunosignal subcellular localization by counting 100 cells per coverslip.

### Western blotting.

For infection studies using whole-cell lysates, cells were washed with PBS, harvested, centrifuged at 10,000 × *g* for 10 min, and lysed in radioimmunoprecipitation assay buffer (50 mM Tris-HCl [pH 7.4], 150 mM NaCl, 1% NP-40, 1% sodium deoxycholate, 1 mM EDTA [pH 8]) containing Halt protease and phosphatase inhibitor cocktail (Thermo Fisher Scientific). Protein lysate concentrations were determined using a Bradford assay (Bio-Rad). Equivalent amounts of lysates were resolved by SDS-PAGE in 4 to 15% TGX polyacrylamide gels (Bio-Rad) at 110 V for 15 min, followed by 200 V for 25 min. Proteins were transferred onto nitrocellulose membrane in Towbin buffer at 100 V for 30 min. Blots were blocked and probed with either 5% (vol/vol) nonfat dry milk or 5% (vol/vol) BSA in Tris-buffered saline plus 0.05% Tween 20 (TBS-T) and then were screened with rabbit or mouse anti-Flag (Sigma-Aldrich [catalog number F7425 or F1804], 1:1,000), rabbit anti-lamin A/C (Cell Signaling, Danvers, MA [2032S]; 1:1,000), mouse anti-GAPDH (Santa Cruz, Dallas, TX [sc-365062]; 1:750), rat anti-Ank13 at 1:1,000, rabbit anti-TSA56 (27; 1:1,000), rat anti-NLRC5 (MilliporeSigma [MABF260]; 1:1000), rabbit anti-Cul1 (Abcam, Cambridge, United Kingdom [ab75817]; 1:1,000), rabbit anti-Skp1 (Cell Signaling [2156S]; 1:750), and rabbit anti-Rbx1 (Abcam [ab133565]; 1:1,000). Bound primary antibodies were detected using horseradish peroxidase-conjugated horse anti-mouse, anti-rabbit, or anti-rat IgG (Cell Signaling Technology; 1:10,000). All blots were incubated with either SuperSignal West Pico PLUS, SuperSignal West Dura, or SuperSignal West Femto chemiluminescent substrate (Thermo Fisher Scientific) prior to imaging in a ChemiDoc Touch Imaging System (Bio-Rad). Bio-Rad Image Lab 6.0 software was used to obtain densitometric values.

### Cytoplasmic and nuclear fractionation.

Transfected or O. tsutsugamushi-infected HeLa cells were washed with PBS, harvested, and lysed following the nuclear fractionation kit (Abcam) protocol. In a singular case when it was necessary to increase endogenous NLRC5 levels prior to fractionation, HeLa cells were stimulated with 20 ng ml^−1^ human IFN-γ (PeproTech, Rock Hill, NJ) for 18 h. To inhibit nuclear import, 3 h prior to collection (16 h posttransfection), spent medium was replaced with fresh media containing either 50 μM importazole (Sigma-Aldrich) or DMSO as a vehicle control prior to nuclear fractionation. Cytoplasmic and nuclear fraction lysates were resolved by SDS-PAGE and subjected to Western blot analysis.

### Immunoprecipitation.

Transfected HeLa cells were harvested and lysed in high saline Tris buffer (50 mM Tris HCl, 400 mM NaCl, 1 mM EDTA [pH 7.4]) with 1.0% Triton x-100 (TBHS-T) spiked with Halt protease and phosphatase inhibitor cocktail (Thermo Fisher Scientific). Protein A/G agarose beads (Thermo Fisher Scientific) were washed with TBHS-T buffer three times, centrifuged at 8,400 × *g* for 30 s, and added to normalized cell lysates in a final volume of 400 μl. The samples were rotated with beads at 4°C for 4 h, followed by centrifugation at 8,600 × *g* for 30 s. Recovered supernatants were added to Anti-Flag M2 affinity gel (MilliporeSigma) that had been washed with TBHS-T buffer three times. Samples were rotated with beads at 4°C overnight followed by centrifugation at 8,600 × *g* for 30 s and washing with TBHS-T 6 to 10 times. Washed beads were resuspended in Laemmli buffer and incubated at 100°C for 5 min to elute bound proteins. Inputs (30 μg) and eluates were resolved by SDS-PAGE and screened by Western blotting.

### RNA isolation and RT-qPCR.

Total RNA was isolated from synchronously infected cells at 2, 4, 8, 24, 48, and 72 h using the RNeasy minikit (Qiagen). Then, 1 μg RNA was treated with amplification-grade DNase (Invitrogen). cDNA was generated using the iScript reverse transcription supermix protocol (Bio-Rad). To verify successful genomic DNA depletion, parallel reactions performed in the absence of reverse transcriptase were used as the template for PCR with human *GAPDH*-specific primers (19) and MyTaq polymerase (Bioline, Taunton, MA) as described above. qPCR using cDNA as the template was performed with SsoFast EvaGreen supermix (Bio-Rad) and *GAPDH*, O. tsutsugamushi 16S rDNA (*ott16S*) (29), and *ank13* primers (29). Thermal cycling conditions used were 95°C for 30 s, followed by 40 cycles of 95°C for 5 s and 55°C for 5 s. Relative expression was determined using the 2^−ΔΔ^*^C^*^T^ method (73) as part of the CFX Maestro for Mac 1.0 software package (Bio-Rad).

### Yeast toxicity assays.

Inserts encoding Ank13, Ank13_I127R_, and Ank13ΔF-box were cloned into a modified pYesNTA-Kan vector (74) to be in-frame with both the Gal promoter and His-tag. The *ank13* coding sequence was PCR amplified using primers *ank13*+1 KpnI F and *ank13* XbaI R (see Table S2), Platinum *Taq* DNA polymerase High Fidelity (Invitrogen), and pFlag-Ank13 as the template. Thermal cycling conditions were 98°C for 30 s, followed by 25 cycles of 98°C for 10 s, 55°C for 30 s, and 72°C for 2 min, and a final extension at 72°C for 10 min. *ank13ΔF-box* was PCR amplified using primers *ank13*+1 KpnI F and *ank13ΔF-box* XbaI R (see Table S2), Platinum *Taq* DNA polymerase High Fidelity, and pFlag-Ank13ΔF-box as the template. Amplicons were digested with KpnI-HF and XbaI, gel purified, and ligated into pYesNTA-Kan to generate pYes-Ank13 and pYes-Ank13ΔF-box. pYes-Ank13_I127R_ was engineered using pYes-Ank13 as the template, In-Fusion primers Ank13_I127R_ F and Ank13 _I127R_ R (see Table S2) according to the TaKaRa Bio USA In-Fusion mutagenesis protocol. Construct sequence integrity was verified by DNA sequencing (Genewiz). The toxicity of Ank13, Ank13_I127R_, and Ank13ΔF-box in S. cerevisiae W303 was assessed as described previously (74). Briefly, S. cerevisiae was transformed with pYesNTA-Kan constructs for expressing Ank13, Ank13_I127R_, Ank13ΔF-box, C. trachomatis CT694 (75), or empty pYesNTA-Kan. Yeast transformants were plated on uracil dropout media containing glucose. Individual colonies were selected and expanded in uracil dropout broth containing glucose. Toxicity was assessed by dilution to an optical density at 600 nm of 0.2 and spotting 10-fold serial dilutions onto 2% glucose (noninducing conditions) and 2% galactose (inducing expression of the fusion protein) agar plates and incubated at 30°C for 48 h. Images were captured using a UVP GelDoc-It (Analytik Jena, Jena, Germany).

### RNA-seq.

HeLa cells transfected to express GFP-Ank13, -Ank13_I127R_, -Ank13ΔF-box, or GFP were washed with PBS, trypsinized, and recovered by centrifugation at 500 × *g* for 5 min. The resulting cell pellets were resuspended in 1 ml of filtered EDTA (Versene) solution (0.526 mM; Irvine Scientific, Santa Ana, CA) with 2% (vol/vol) heat-inactivated fetal bovine serum (FBS). GFP-expressing cells were sorted from nontransfected cells on a FACSAria Fusion SORP High-Speed Cell Sorter using BD FACSDiva 8.0.1 (BD Biosciences) and collected in 10% (vol/vol) FBS in PBS. Histograms of transfected and nontransfected control cells were generated using FloJo V10 software (BD Biosciences). Total RNA isolated from triplicate samples of each sorted population and HeLa cells that had been synchronously infected with O. tsutsugamushi for 4 h or 48 h were submitted to Novogene (Sacramento, CA) for RNA-seq analyses as follows. Sample integrity was confirmed using an Agilent 2100 Bioanalyzer (Agilent Technologies, Santa Clara, CA). Reads were mapped to human reference genome (GRCh38/hg38) sequenced to a paired-end 250- to 300-bp insert cDNA library using an Illumina high-throughput sequencing platform (Illumina, San Diego, CA). Alignments were parsed using STAR program. Venn diagram and volcano plot analyses were achieved through differential expression significant analysis of two conditions or groups using the DESeq2 R software package 1.14.1 (Bioconducter). Resulting *P* values were adjusted using Benjamini and Hochberg’s approach for controlling the false discovery rate (76). The differential expression criterion was an adjusted *P* value (*P*_adj_) < 0.05 and based on the negative binomial distribution (58). Genes were classified per biological processes by analyzing Gene Ontology (GO) terms (www.geneontology.org). The R package, ClusterProfiler (77), was used for statistical analysis of GO and those terms with a corrected *P* value of <0.05 were considered significantly enriched.

### Bioinformatic analysis.

cNLS Mapper (http://nls-mapper.iab.keio.ac.jp/cgi-bin/NLS_Mapper_form.cgi) (52) was used to predict importin α-dependent nuclear localization signals. STRING (59) was used to analyze protein-protein interaction networks (https://string-db.org/cgi/input.pl).

### Statistical analysis.

Statistical analyses were performed using the Prism 8.0 software package (GraphPad, San Diego, CA). One-way analysis of variance (ANOVA) with Tukey’s *post hoc* test was used to test for a significant difference among groups. Two-way ANOVA with Dunnett’s correction was used to assess for significant differences among the percentages of cells exhibiting cytoplasmic, throughout the cell, or nuclear localization of Flag-tagged proteins. Statistical significance was set at *P* values of <0.05.
