# Supplementary material for: Orientia tsutsugamushi Nucleomodulin Ank13 Exploits the RaDAR Nuclear Import Pathway To Modulate Host Cell Transcription
Source: mBio. 2021 Aug 3;12(4):e01816-21. doi: 10.1128/mBio.01816-21 (PMC8406279; doi:10.1128/mBio.01816-21)
Supplement: TABLE S1 [file mbio.01816-21-st001.docx]

Table S1. Comparison of full-length Ank13 homologs in other *O. tsutsugamushi* strains/isolates

| Strain/ isolate | Geographic Origin | Reference | Nucleotide length | % Nucleotide Identity | Protein Accession  Number | AA Length | % AA  Identity | % AA  Similarity | Number of ARs | 13th residue of AR4 |
| --- | --- | --- | --- | --- | --- | --- | --- | --- | --- | --- |
| Ikeda | Japan | Tamura 1984 | 1470 | 100 | BAG40310 | 490 | 100 | 100 | 8 | I |
| Kato | Japan | Shishido 1958 | 1473 | 92 | SPR12256.1 | 491 | 81 | 87 | 8 | V |
| UT76 | NE Thailand | Blacksell  2008 | 1473 | 89 | SPR05556.1 | 491 | 78 | 85 | 8 | V |
| UT176 | NE Thailand | Luksamee-tanasan  2007 | 1473 | 89 | SPR09063.1 | 491 | 77 | 85 | 8 | V |
| Wuj/2014 | China |  | 1473 | 89 | QES95870.1 | 491 | 77 | 85 | 8 | V |
