# Supplementary figures and images for: Orientia tsutsugamushi Nucleomodulin Ank13 Exploits the RaDAR Nuclear Import Pathway To Modulate Host Cell Transcription
Source: mBio. 2021 Aug 3;12(4):e01816-21. doi: 10.1128/mBio.01816-21 (PMC8406279; doi:10.1128/mBio.01816-21)

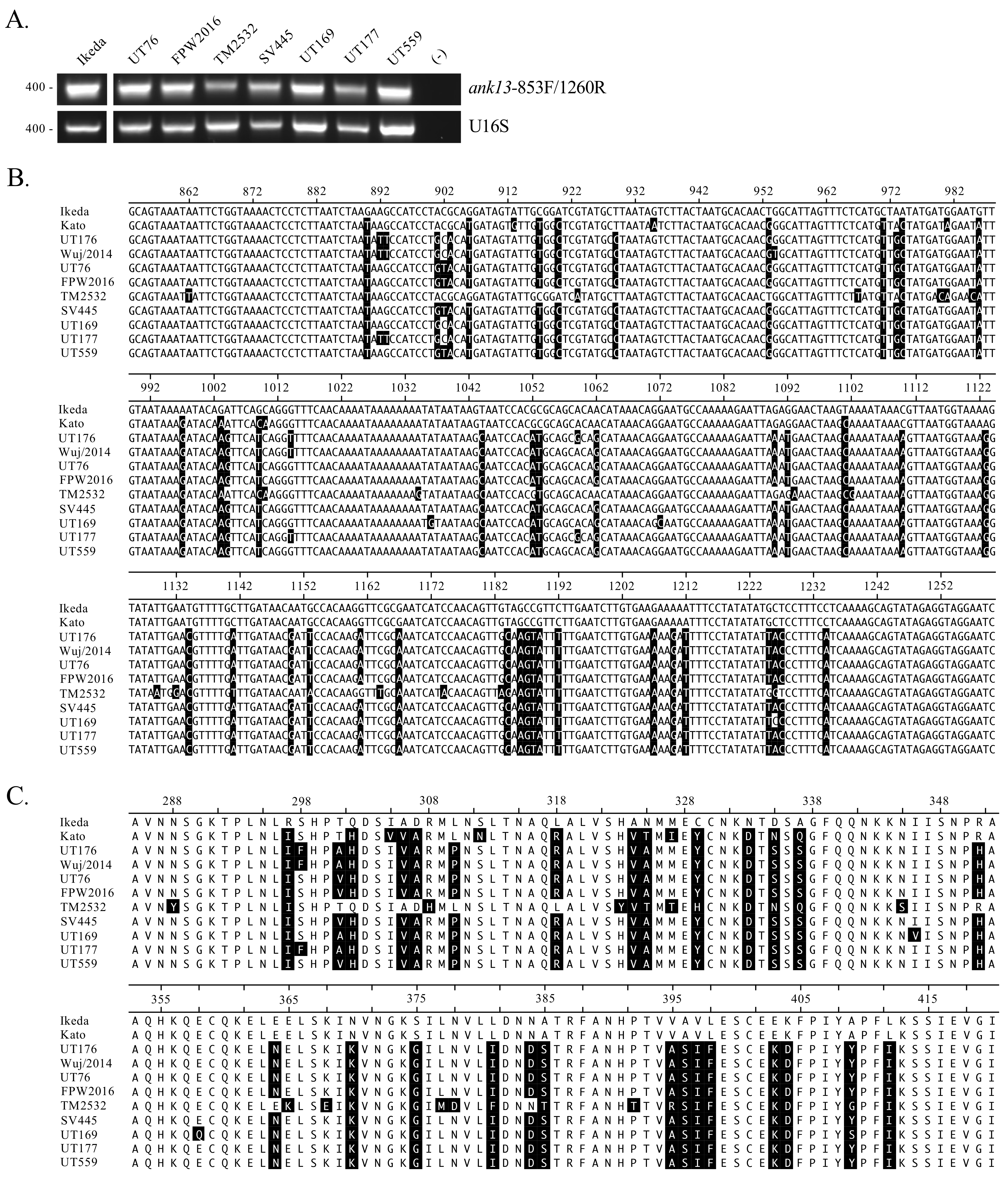

Supplement: FIG S1 [file mbio.01816-21-sf001.tiff]

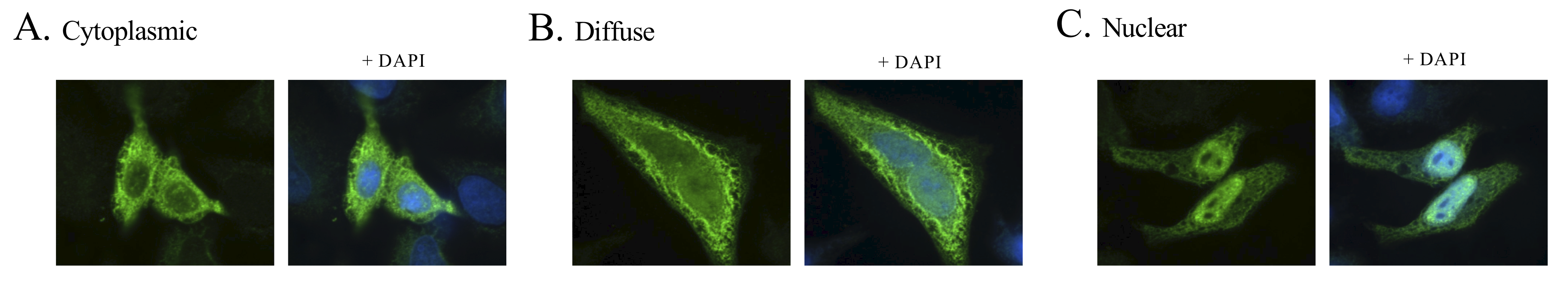

Supplement: FIG S2 [file mbio.01816-21-sf002.tiff]

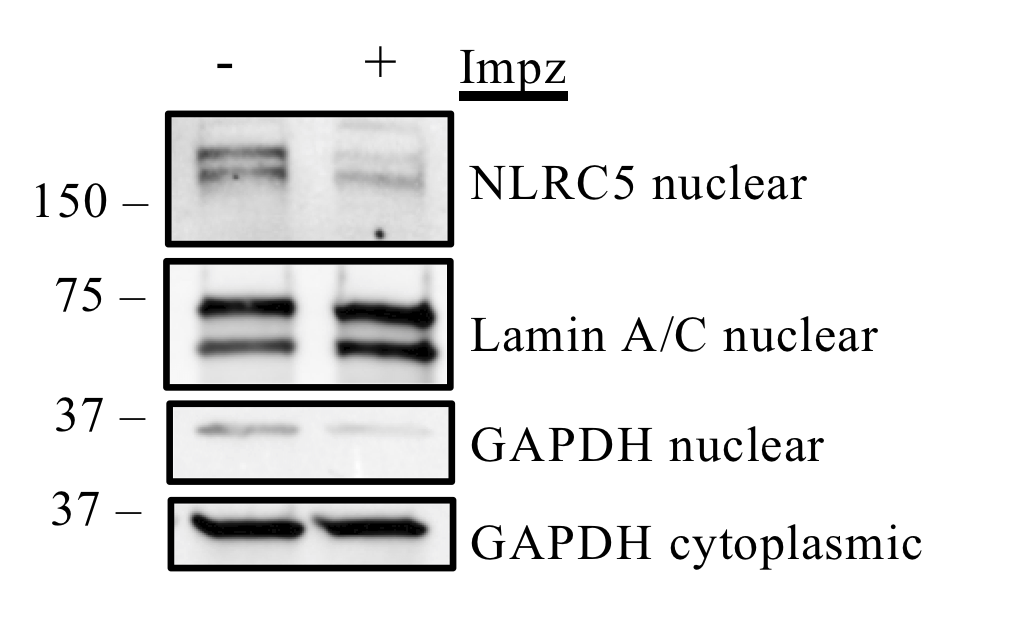

Supplement: FIG S3 [file mbio.01816-21-sf003.tiff]

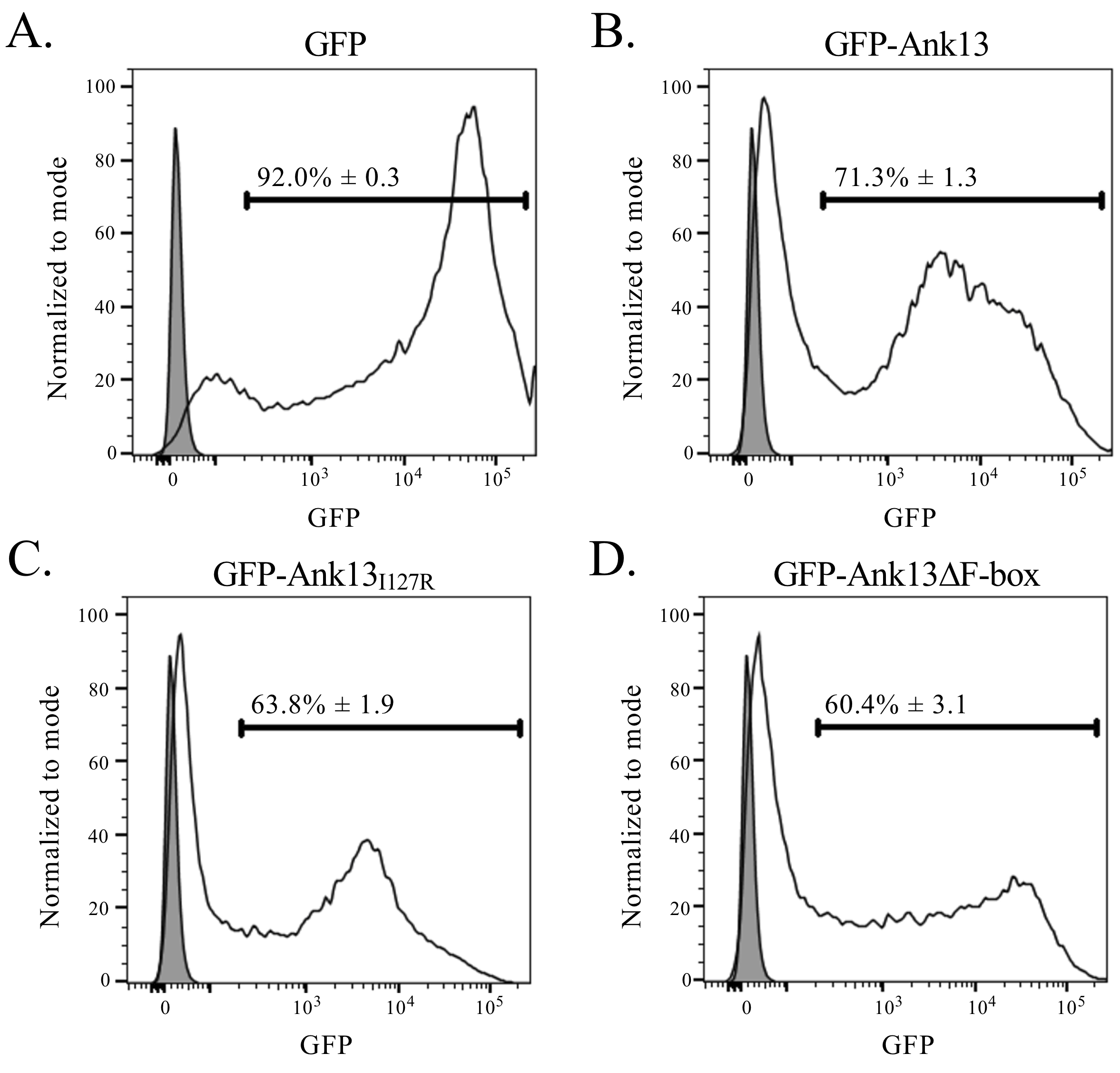

Supplement: FIG S4 [file mbio.01816-21-sf004.tiff]

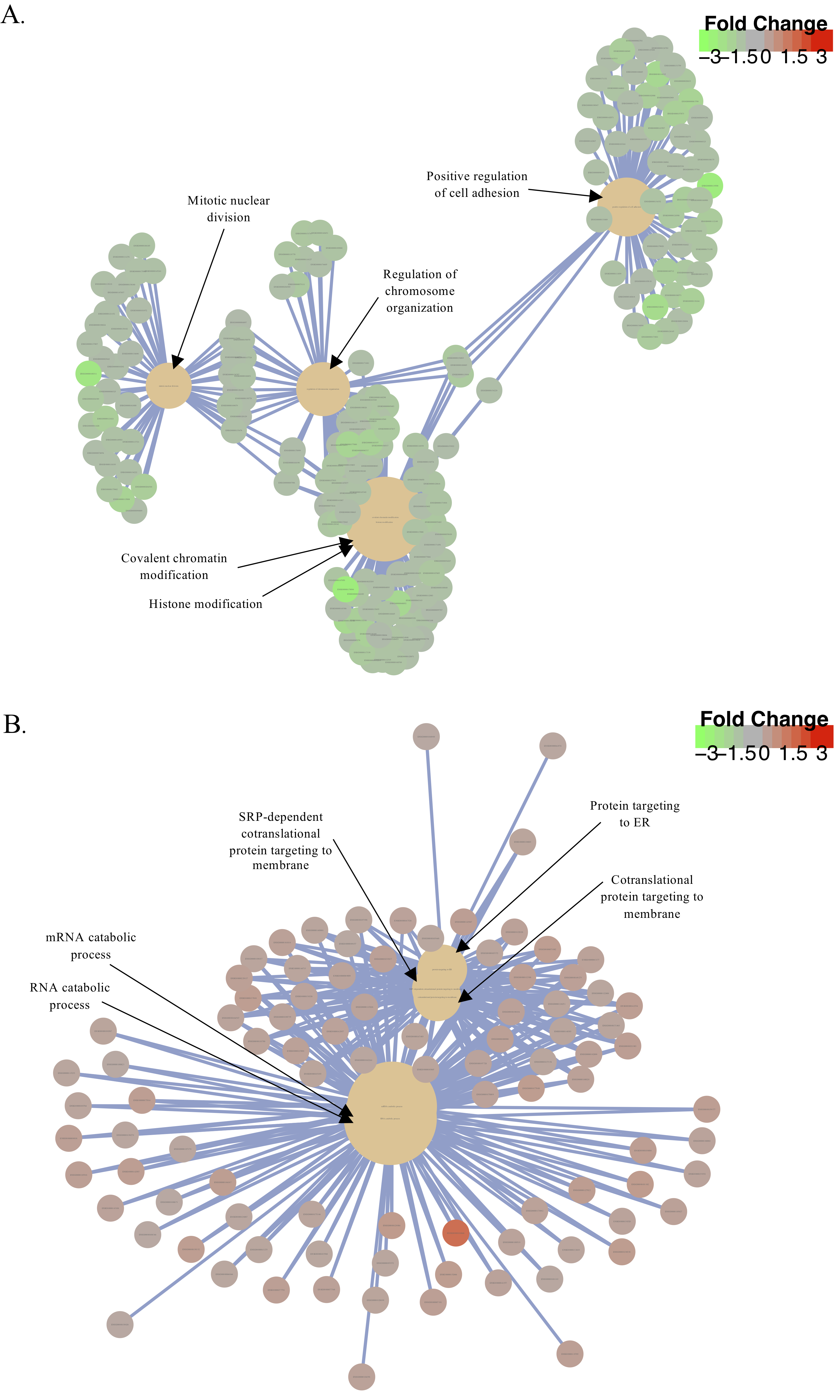

Supplement: FIG S5 [file mbio.01816-21-sf005.tiff]
