# Supplementary material for: Orientia tsutsugamushi Nucleomodulin Ank13 Exploits the RaDAR Nuclear Import Pathway To Modulate Host Cell Transcription
Source: mBio. 2021 Aug 3;12(4):e01816-21. doi: 10.1128/mBio.01816-21 (PMC8406279; doi:10.1128/mBio.01816-21)
Supplement: TABLE S2 [file mbio.01816-21-st002.docx]

Table S2. Oligonucleotides utilized in this study

| Designation*^a^* | Sequence (5’-3’) |
| --- | --- |
| *ank13*-853F  *ank13*-1260R  Flag-Ank13_49-490_ F*^b^*  Flag-Ank13_49-490_ R*^b^*  Flag-Ank13∆F-box F*^b^* | GCAGTAAATAATTCTGGTAAAACTCCTC  GATTCCTACCTCTATACTGCTTTTG  *TTGCGGCCGCGAATTCT*TTGGACGATGGTGGCGA  *ATGCCACCCG*GGATCCTCAGATGCCGAGACTTGAAGAT  *TTGCGGCCGCGAATTCT*GCCCCTTTCTCTACCAAA |
| Flag-Ank13∆F-box R*^b^* | *ATGCCACCCG*GGATCCTCACACGTTCCAGTAATTGC |
| Ank13_V62R_ F*^c^* | CGCTGTGCGAAATGGGCAGGTGGAGGTGG |
| Ank13_V62R_ R*^c^* | CCATTTCGCACAGCGTGGTACAGGCAGG |
| Ank13_A95R_ F*^c^* | CGCGGTGCGAAACGGCCAGAAGGACATCGTC |
| Ank13_A95R_ R*^c^* | CCGTTTCGCACCGCGAAACAGATGCAG |
| Ank13_I127R_ F*^c^* | TTATGCCCGAGCTAACCGGAACACCGACA |
| Ank13_I127R_ R*^c^* | TTAGCTCGGGCATAATGGAGGATTGGTATGCT |
| Ank13_I127L_ F*^c^* | TTATGCCCTGGCTAACCGGAACACCGACA |
| Ank13_I127L_ R*^c^* | TTAGCCAGGGCATAATGGAGGATTGGTATGCT |
| Ank13_I161R_ F*^c^* | CGCTTGCCGAGGAAAGGGCATAAACTTGGAA |
| Ank13_I161R_ R*^c^* | TTTCCTCGGCAAGCGTAGTGAAGGGTG |
| Ank13_I161L_ F*^c^* | CGCTTGCCTGGGAAAGGGCATAAACTTGGAA |
| Ank13_I161L_ R*^c^*  *ank13* +1 KpnI F*^d^*  *ank13* XbaI R*^d^*  *ank13∆*F-box XbaI R*^d^* | TTTCCCAGGCAAGCGTAGTGAAGGGTG  **CGACGATGACGATAA**GGTACCCATGGCCCCTTTCTCTACCAAAGACG  **CCTTCGAAGGGCCC**TCTAGATCAGATGCCGAGACTTGAAGATTTATTCTTGTTC  **CCTTCGAAGGGCCC**TCTAGATTACACGTTCCAGTAATTGCGTGGAG |

*^a^*F and R refer to primers that bind to the sense and antisense strand, respectively.

*^b^*Italicized text indicate nucleotides complementary to the p3XFLAG-CMV-7.1 vector.

*^c^*Highlighed text indicate nucleotides used to make site-directed mutants.

*^d^*Boldface text indicates nucleotides complementary to the pYesNTA-Kan plasmid. Restriction sites are underlined.
